# Multimodal mass spectrometric characterization of structural microheterogeneity in rituximab reference and biosimilars

**DOI:** 10.1016/j.ijbiomac.2025.149062

**Published:** 2025-11-17

**Authors:** Youngseo Na, Jill L. Kinzer, Luke Morrissette, Young Seok Cho, Brian Shay, Michael Ford, Anna Schwendeman

**Affiliations:** a Department of Medicinal Chemistry, College of Pharmacy, University of Michigan, 428 Church St, Ann Arbor, MI, 48109, USA; b Department of Pharmaceutical Sciences, College of Pharmacy, University of Michigan, 428 Church St, Ann Arbor, MI, 48109, USA; c MS Bioworks, 3950 Varsity Dr, Ann Arbor, MI, 48108, USA; d Biointerfaces Institute, NCRC, 2800 Plymouth Rd, Ann Arbor, MI, 48109, USA; e Department of Microbiology, School of Medicine, Sungkyunkwan University, Suwon, Republic of Korea

**Keywords:** Rituximab, Post-translational modifications (PTMs), Mass spectrometry

## Abstract

Three biosimilars to Rituxan^®^ (rituximab)—Truxima^®^, Ruxience^®^, and Riabni^™^—have received FDA approval. Monoclonal antibodies exhibit inherent heterogeneity due to post-translational modifications, and defining acceptable ranges of such variability remains a critical challenge in biosimilar development. While pairwise comparisons between a biosimilar and its reference product are extensively studied, comprehensive analyses across multiple products are limited. In this study, we systematically characterized Rituxan^®^ and its three FDA-approved biosimilars using advanced mass spectrometry (MS)-based techniques. Native intact MS assessed molecular weight variations, LC-FLR-MS glycan profiling evaluated glycoform distributions, and LC-MS/MS peptide mapping examined sequence integrity and modifications. Notably, the greatest glycan-related variation was observed between biosimilars—between Ruxience^®^ and Truxima^®^ in glycoform distribution, and between Ruxience^®^ and Riabni^™^ in total afucosylated glycan levels—which correlated with slight differences in in vitro antibody-dependent cellular cytotoxicity activity. However, overall heterogeneity between biosimilars and the reference product was relatively small compared to that observed among biosimilars, with results consistent across analytical methods. Since commercially available biosimilars were analyzed, the observed variations reflect real-world analytical variability that does not impact clinical performance. These findings, which offer detailed analytical comparisons of biosimilars, provide insights into acceptable structural variation and support efforts to refine regulatory assessment practices.

## 1. Introduction

As patents on therapeutic monoclonal antibodies (mAbs) expire, the development and commercialization of biosimilars begins to rise. Emergence of biosimilars has contributed to increased affordability of therapeutic biologics by enhancing competition and improving patient access [1]. To date, the FDA has licensed 69 biosimilar products and 13 interchangeable products [2].

The manufacturing of mAbs is a complex process involving multiple production and purification bioprocessing steps that often result in mAb heterogeneities known as chemical/post-translational modifications (PTMs). These modifications, which can affect drug’s stability, immunogenicity, and efficacy, are considered critical quality attributes (CQAs) [3]. As such, the abundance of CQAs including PTMs must be carefully monitored throughout the product development, marketing, and post-approval phases [4].

Physicochemical differences arising from PTMs play a crucial role in the overall therapeutic activity and safety by mechanisms such as altering the binding affinity of IgG antibodies for the neonatal Fc receptor (FcRn) or the FcγIIIa receptor [5,6]. For instance, N-glycosylation, which is omnipresent on the Fc portion of therapeutic IgG1 mAbs, is one of the most important CQAs as it contributes to the structural integrity and conformation of the Fc region [7,8]. Moreover, distinct N-glycosylation types, such as afucosylated or sialylated glycoforms, can modulate the binding affinity of the mAb to FcγIIIa, and hence, alter mAbs’ antibody dependent cellular cytotoxicity (ADCC) [6,9,10]. Additionally, chemical modifications on amino acids, such as oxidation, deamidation, and isomerization, in the Fab and Fc domains, can impair the binding to the target epitope and to the FcRn, which may impact drug efficacy and clearance [11,12].

Heterogeneities in a mAb population are often unavoidable, even within singular lots of the same therapeutics produced by a company. For biosimilar developers, who have limited knowledge of the innovator product’s manufacturing processes, these challenges can be amplified. The development of biosimilars is grounded in the analytical comparison of a proposed biosimilar product to its reference product (or the innovator product), with biosimilar developers striving to demonstrate that their product is highly similar to and has no clinically meaningful differences from the reference product. Therefore, to ensure biosimilarity, rigorous comprehensive protein characterization is needed throughout the development and manufacturing processes. Several analytical methods are employed for this purpose, including intact mass spectrometry (MS), tandem mass spectrometry (LC-MS/MS), and released glycan analysis via liquid chromatography.

In early 2023, the FDA introduced the BsUFA III regulatory research pilot program with goals of advancing the development of biosimilar development and improving regulatory efficiency. Through this program, the FDA is focused on standardizing the assessment and reporting of product quality attributes, characterizing relationships between product quality attributes and clinical outcomes, and improving new analytical technologies for protein characterization [13]. These commitments align with the evolving perspective that a robust analytical and functional similarity assessment could be the foundation for regulatory decisions regarding biosimilar mAbs [14]. This is particularly relevant given that comparative clinical studies of biosimilars are generally less sensitive than analytical studies to differences between products, and confidence in the safety and efficacy of biosimilars is growing [15,16]. Recent FDA guidance indicates that when residual uncertainty can be addressed through analytical and functional data, the need for separate clinical efficacy comparisons may be reduced [17,18]. This reflects a regulatory shift toward greater reliance on analytical data as the foundation for biosimilar approval.

Rituximab is a chimeric human/murine mAb that targets CD20 on the surface of malignant B cells, and it is licensed for the treatment of non-Hodgkin’s lymphoma, chronic lymphocytic leukemia, and refractory autoimmune disorders [19,20]. Rituxan^®^ (or MabThera^®^ in Europe) is the reference product, developed by Genentech/Roche, which was approved by the FDA in 1997 [21]. Following the expiration of its patent in the US in 2018 and in Europe in 2013, many companies have pursued the development of rituximab biosimilars. To date, FDA has approved three rituximab biosimilars, which are produced by Celltrion Healthcare (Truxima^®^, FDA approved in November 2018), Pfizer (Ruxience^®^, FDA approved in July 2019), and Amgen (Riabni^™^, FDA approved in December 2020).

Here, we evaluated the physicochemical heterogeneity of multiple batches of the reference product rituximab (Rituxan^®^) and all three FDA-approved biosimilars (Truxima^®^, Ruxience^®^, and Riabni^™^). We utilized three advanced MS-based techniques—intact MS, released glycan analysis, and peptide mapping—which are commonly used in Biologic License Application (BLA) submission. These methods allowed for an in-depth characterization of PTMs, including N-glycosylation, N-terminal pyroglutamic acid (pyroGlu) formation, C-terminal lysine clipping, methionine and tryptophan oxidation, and deamidation and succinimidation on asparagine and glutamine residues. Furthermore, we assessed the ADCC activity to see how PTMs variations impact rituximab’s mechanisms of action (MOAs) [22].

While biosimilar approvals are generally accompanied by publications summarizing the comparative analytical assessment data from biosimilar developers including modifications that may influence the functionality and safety of the mAb [7,8], few publications authored by independent analytical laboratories have evaluated biosimilars separately from the developer’s [23,24]. In contrast to previous comparative studies that evaluate between a reference product alongside a single biosimilar or biosimilar candidate, our study includes the reference and all three FDA-approved biosimilars, evaluated under identical experimental conditions. This provides an unbiased and comprehensive analytical characterization of the physicochemical variations across these products. Moreover, our study aims to characterize the real-world analytical variability observed among FDA-approved rituximab products. By systematically comparing multiple approved biosimilars under identical analytical conditions, we provide data-driven insights that can inform future discussions on defining acceptable ranges of physicochemical variations.

## 2. Materials and methods

### 2.1. Materials

Digestion reagents, including iodoacetamide, TCEP and trypsin with Lys-C were acquired from Promega (Madison, WI). Sample plates for the digestion reaction were purchased through Agilent (Santa Clara, CA). LC-Fluorescence reagents were provided in the purchased Instant PC (Agilent). Rituxan^®^ (Genentech), its FDA approved biosimilars Truxima^®^ (Celltrion, Nov 2019), Ruxience^®^ (Pfizer, Jan 2020) and Riabni^™^ (Amgen, Jan 2021) were examined. For method validation, the NIST mAb, a humanized IgGκ monoclonal antibody, was used as a standard control to ensure that all measurements within the same analytical run were internally consistent and met predefined quality control criteria. The NIST mAb was analyzed within each experimental batch alongside test samples to monitor instrument performance, mass accuracy, and retention time stability. NIST mAb was provided by NIIMBL in 800 μL aliquots of 10 mg/mL mAb in its native formulation buffer. Rituxan^®^ (rituximab, lot#: 3491321, 3575428, and 3,580,141), Truxima^®^ (rituximab-abbs, lot#: 0 L0031, 1B1011, and 1E11001), Ruxience^®^ (rituximab-pvvr, lot#: FC5021, GN4445, and GY2269), and Riabni^™^ (rituximab-arrx, lot#: 1128511, 1141263, and 1,157,031) were procured from the University of Michigan hospital pharmacy (10 mg/mL) and stored at 4 °C until use.

### 2.2. Intact MS

Intact mass antibody characterization was performed in 2 different ways; without enzyme treatment and deglycosylated. For deglycosylated intact mass: 20 μg of antibody was incubated with 3 μL PNGase F (Promega) in 50 mM ammonium bicarbonate at 37 °C for 3 h. Samples were diluted to 1 pmol/μL with water. Chromatographic analysis was performed on an Acquity M Class HPLC (Waters, Milford, MA), interfaced with a Q-Exactive mass spectrometer (ThermoFisher, Waltham, MA) and employed a XBridge Protein BEH C4 column (300 Å, 3.5 um, 2.1 mm × 50 mm; Waters, Milford, MA). 5 pmol of digested or native protein were loaded onto the column via a 5 μL injection. Separation was performed over a 9-min gradient with an increasing percentage of mobile phase B from 2 % to 20 % at a flow rate of 50 μL/min. Mobile phase A was 0.1 % formic acid in water and mobile phase B was 0.1 % formic acid in acetonitrile. The column temperature was maintained at 60 °C. Mass analysis was performed in full scan, positive ion mode with an in-source CID at 45 eV. The scan range was 1000–4000 *m*/*z* with 10 microscans per second with the resolution of 17,500, ACG target of 3 × 10^6^ and max IT of 250 ms. Spectral deconvolution was performed with Byos Intact workflows (Protein Metrics Inc., Cupertino, CA).

### 2.3. Released glycan assay

The N-glycans from proteins were released from the antibody and analyzed through MS. N-glycans were prepared from antibody samples (15 μg) using GlyX N-glycan prep with InstantPC kit (Agilent, Santa Clara, CA) according to the manufacturer’s protocol. Due to buffer incompatibility with the kits, NIST mAb and rituximab samples (Rituxan^®^, Truxima^®^, Ruxience^®^, and Riabni^™^) were buffer exchanged into water using 0.5 mL, 10 kDa MWCO Amicon spin filters and further concentrated to 2 mg/mL. The final protein concentrations were measured using the protein A_280_ IgG setting on a NanoDrop (ThermoFisher). 40 μg of each glycoprotein sample was used for the assay. N-Glycans were released from N-Glycanase as their glycosylamines and labeled with instant PC followed by HILIC-FLR-Q-ToF analysis. The LC-FLR-MS method was based on the vendor recommended method provided for each kit. The instrument system was a Waters Acquity H Class HPLC interfaced with a Waters fluorescence detector and a Waters Xevo G2-XS Q-ToF. The column used for all kits and samples was a Waters ACQUITY UPLC Glycan BEH Amide, 130 Å, 1.7 μm, 2.1 × 150 mm column kept at 60 °C. The flow rate was 0.4 mL/min over the 60-min run time. Labeled samples were injected at 1 μL and separation was achieved using a gradient method starting at 25 % aqueous phase raising to 100 %. Mobile phase A was 50 mM ammonium formate pH 4.4 and mobile phase B was 100 % acetonitrile. For InstantPC, the fluorescence excitation wavelength was 285 nm and emission wavelength was 345 nm. For all samples, the following Q-ToF parameters were used: positive mode, capillary voltage 2.8 kV, cone voltage 30 V, source temperature 120 °C, desolvation temperature 350 °C, scan time 0.8 s, and *m*/*z* range 300–2000 Da.

Analysis of the released glycans LC-MS data was performed using ProteinMetrics Byos software with Released glycan analysis workflows. Glycan identity was determined by the m/z values obtained for each sample.

### 2.4. Peptide mapping

Protein Digestion. To minimize artifacts formed during the protein digestion process, we conducted a low-pH peptide-mapping method under mildly acidic conditions using an AccuMAP Low pH Protein Digestion kit (Promega Inc.). To ensure a fair comparison and minimize potential errors from sample handing and individual variations in analysis, all samples were analyzed under identical conditions at the same time. The digestion process was conducted using an AssayMAP Bravo liquid handling platform (Agilent) to reduce handling errors, minimize potential operator biases, and ensure consistent processing conditions across all samples. Samples were prepared following the vendor’s AccuMAP Low pH Protein Digestion protocol for 5 mg/mL protein samples. The concentration of iodoacetamide was changed from 300 mM to 100 mM and increasing the volume of the iodoacetamide added from 2 μL to 6 μL per sample. This adjustment was made to accommodate the AssayMAP Bravo pipetting volume limitations. Following an addition of low-pH-resistant Lys-C and modified trypsin to cleave at basic amino acid residues (K and R) samples were incubated at 37 °C overnight. Samples were then acidified with 20 % TFA and purified using C18 solid phase extraction cartridges on the AssayMAP Bravo. For purification, the equilibration/utility buffer was 0.1 % formic acid, and the priming/syringe wash and elution buffers were 80 % ACN in 0.1 % FA. For each sample, 50 μL was eluted at a flow rate of 5 μL/min. The peptide concentration of each sample was measured using a Pierce Fluorometric Quantitative Peptide Assay (ThermoScientific, Waltham, MA). The samples were then normalized to 0.1 μg/μL using the AssayMAP Bravo with 0.05 % TFA as the diluent. After normalization, 500 ng sample were loaded onto the column for LC-MS/MS analysis.

LC-MS/MS. An Acquity M Class HPLC (Waters, Milford, MA) was interfaced with an Orbitrap Exploris mass spectrometer (ThermoFisher) for peptide mapping data acquisition. The column used was Luna C18 column (Phenomenex, Torrance, CA). Samples were run using a 30-min gradient with 0.1 % formic acid in water as mobile phase A and 0.1 % formic acid in acetonitrile as mobile phase B. The gradient gradually increased from 2 % to 90 % mobile phase B throughout the run before equilibrating at 2 % between samples. 10 μL, equivalent to 500 ng of protein, were injected on the column at room temperature and flowed at a rate of 0.350 μL/min. The Orbitrap was operated in positive ion mode at a resolution of 6000 and scan range of 300–1600 *m*/*z*. The RF lens was set to 40 %, normalized ACG target set to 300 %, mass tolerance set to 10 ppm, intensity threshold set to 5.0E3 and charge state set to 2–6. The HCD collision energy was set at 30 % using a fixed, normalized collision energy mode. Byos PTM workflows were used to process data and identify and quantify modifications. Modifications were quantified by calculating the percentage of modified peptides relative to the wild-type peptides using extracted ion chromatogram (XIC) areas.

### 2.5. Size exclusion chromatography

All samples were diluted to 1 mg/mL with water. 10 μg of antibody samples (10 μL) were injected onto a Waters Acquity UPLC BEH450 SEC column (2.5 μm, 4.6 × 150 mm) attached to an Acquity UPLC H-class (Waters) system. The mobile phase was phosphate buffered saline, pH 7.4 (Gibco, Fisher Scientific). Size separation took place over 13 min with an isocratic flow rate of 0.3 mL/min. UV absorption data was collected at 280 nm.

### 2.6. ADCC reporter assay

To assess biological activity, ADCC Reporter Bioassay was conducted according to the manufacturer’s instruction (Promega). WIL2-S, a CD20 positive human B lymphoblastoma line, were used as target cells, and Jurkat/NFAT-luc + FcγRIIIa, which constitutively expresses FcγRIIIa receptor and nuclear factor of activated T cells (NFAT) luciferase, were used as effector cells. Briefly, 25 μL of WIL2-S cells (1 × 10^6^/mL) (Promega) and 25 μL of Jurkat cells (6 × 10^6^/mL) (Promega) in logarithmic growth phase were seeded in a 96-well plate. 25 μL of assay buffer containing a therapeutic mAb was added at varying concentrations, prepared by 1:3 serial dilutions starting from 1 μg/mL down to 0.000152 μg/mL. PNGase F treated antibody samples were prepared by 1:3 serial dilutions from 4 μg/mL to 0.610 ng/mL. Rituxan^®^ (lot: 3575428), Truxima^®^ (lot: 1B1011), Ruxience^®^ (lot: GN4445), and Riabni^™^ (lot: 1141263) samples were used for the assay. After incubation at 37 °C in a humidified 5 % CO_2_ for 6 h, luciferase activity was measured using Bio-Glo^™^ Luciferase Assay Reagent (Promega) using a GloMax Explorer plate reader (Promega). Luminescence reflects FcγRIIIa activation in effector cells as a surrogate measure of ADCC activity. Each sample’s fold of induction was calculated by dividing RLU (induced-background) by the RLU (no antibody control - background), and GraphPad Prism was used to graph and interpret the EC_50_ data.

### 2.7. FcγRIIIa binding assay

The interaction of the Fc regions with FcγRIIIa for rituximab samples were measured by Lumit^®^ FcγRIIIa (V158)Binding Immunoassay (Promega) according to the manufacturer’s instruction [25]. Untreated and PNGaseFtreated samples were prepared by 1:3 serial dilutions from 0.75 mg/mL to 0.127 μg/mL. Rituxan^®^ (lot: 3575428), Truxima^®^ (lot: 1B1011), Ruxience^®^ (lot: GN4445), and Riabni^™^ (lot: 1141263) samples were used for the assay. Normalized luminescence was calculated as a percentage of the maximum bioluminescent signal (observed in the absence of an analyte) The dose response curve was fitted with a 4-parameter model using GraphPad Prism^®^ software to calculate IC_50_.

### 2.8. Complement-dependent cytotoxicity (CDC) assay

For CDC assay, WIL2-S cells were seeded in triplicate 96-well plates at 2.5 × 10^4^ cells/well in RPMI 1640 without serum. A therapeutic mAb was added at varying concentrations, prepared by 1:4 serial dilutions from 16 μg/mL to 0.061035 ng/mL (90 μL total volume). Rituxan^®^ (lot: 3575428), Truxima^®^ (lot: 1B1011), Ruxience^®^ (lot: GN4445), and Riabni^™^ (lot: 1141263) samples were used for the assay. After incubation at 37 °C in a humidified, 5 % CO_2_ incubator for 30 min, normal human serum (NHS, Complement Technology, Inc.) was added at 10 %, mixed by pipetting up and down, and then incubated at 37 °C for 90 min. Viability was subsequently evaluated with the CellTiter^®^ 96 Aqueous One Solution (Promega) according to the manufacturer’s instructions. Plates were read on a Spectra Max (Molecular Devices) plate reader for absorbance at 490 nm. Cell viability as a percentage was calculated as: (experimental maximum/spontaneous maximum) × 100. The dose response curve was fitted with a 4-parameter model using GraphPad Prism^®^ software to calculate EC_50_.

## 3. Results

### 3.1. Intact mass analysis

**Fig. 1.**
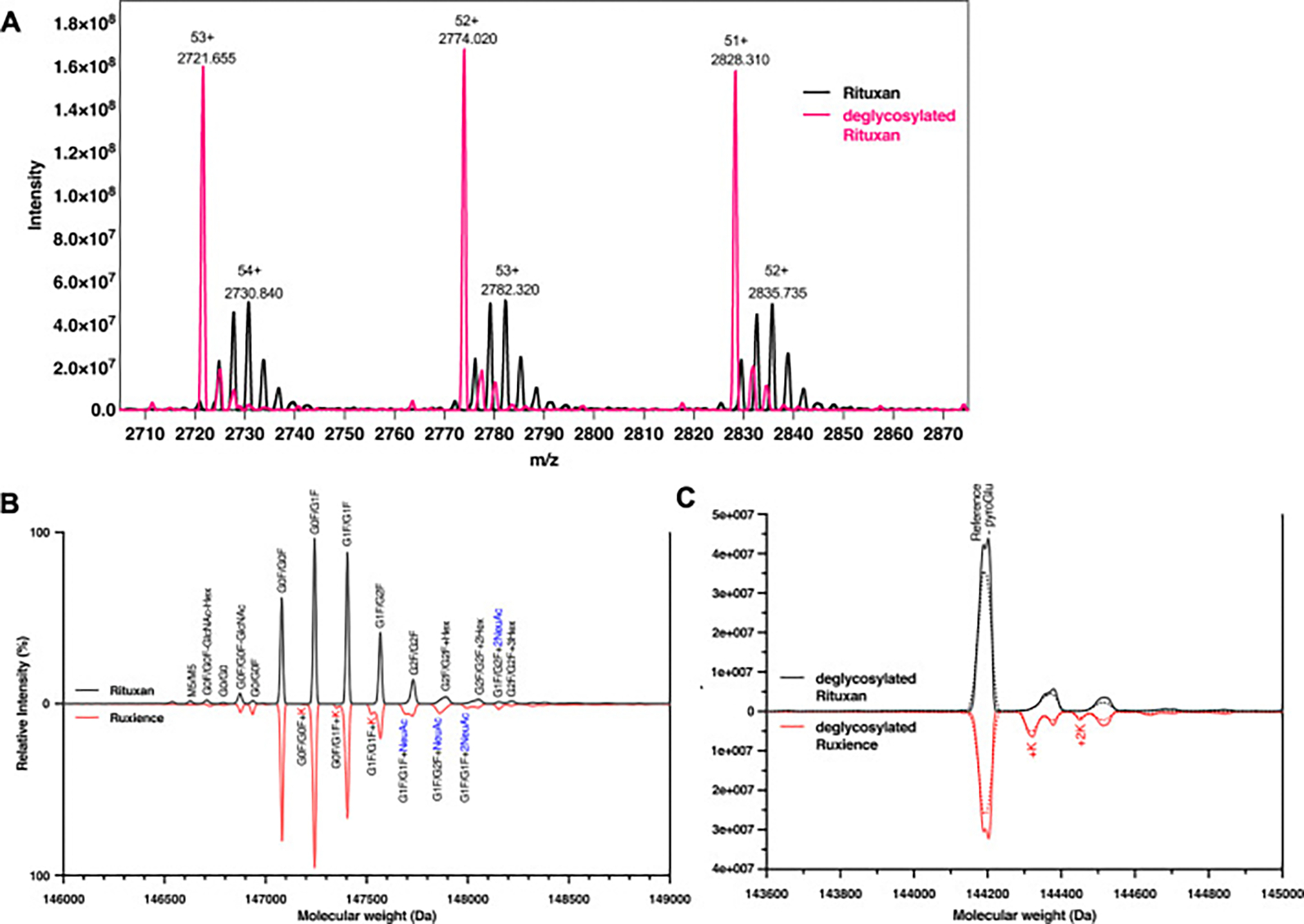
Intact MS analysis under native and deglycosylated conditions. (A) Deconvoluted ESI-Q-ToF MS of intact untreated (black) and PNGase F-treated (pink) Rituxan. PNGase F treatment induces a mass shift and reduces glycoform heterogeneity. Representative mirror plot of the deconvoluted mass spectra of (B) fully glycosylated and (C) deglycosylated by PNGase F treatment Rituxan (black) and Ruxience (red). Differences in relative abundances of glycoforms can be observed between Rituxan and Ruxience. Variant forms having K attached to the C-terminal heavy chain of rituximab were detected in Ruxience. Unsharpened graphs were shown in dotted line and sharpen graphs in solid line. Reference peak means deglycosylated mAb with 4 pyroGlu formation and 2 K clipping. –pyroGlu peak means deglycosylated mAb with 3 pyroGlu formation and 2 K clipping.

Primary structure was first assessed via intact mass measurement via LC-ESI-MS, allowing for the confirmation of theoretical molecular mass, including major PTMs (e.g., glycosylation, C-terminal Lys clipping, N-terminal Gln cyclization, and glycation). The intact masses of reference and biosimilar rituximabs were acquired in their both fully glycosylated and deglycosylated forms. The obtained total ion chromatogram (TIC) profile revealed similar retention time of 4.5 min for both glycosylated and deglycosylated samples (Fig. S1). The three predominant charge states observed in glycosylated antibodies were z = +52, +53 and +54, whereas the charge states in deglycosylated form shifted to z = +51, +52, and + 53. Deconvoluted ESI mass spectra of intact glycosylated and deglycosylated Rituxan are presented in Fig. 1a.

Both heavy (HC) and light (LC) chains of product possess an N-terminal glutamine (Q1), which is prone to cyclization, forming N-terminal pyroGlu (−17.0265 Da). Additionally, the C-terminal lysine (K451) in the HC is susceptible to clipping, resulting in a mass decrease of 128.2 Da. The expected mass of rituximab, computed from its primary amino acid sequences using the intact workflows of Byos, ProteinMetrics, was based on complete disulfide bridge connectivity, N-terminal Gln cyclization, and C-terminal Lys clipping, along with N-linked glycosylation.

**Fig. 2.**
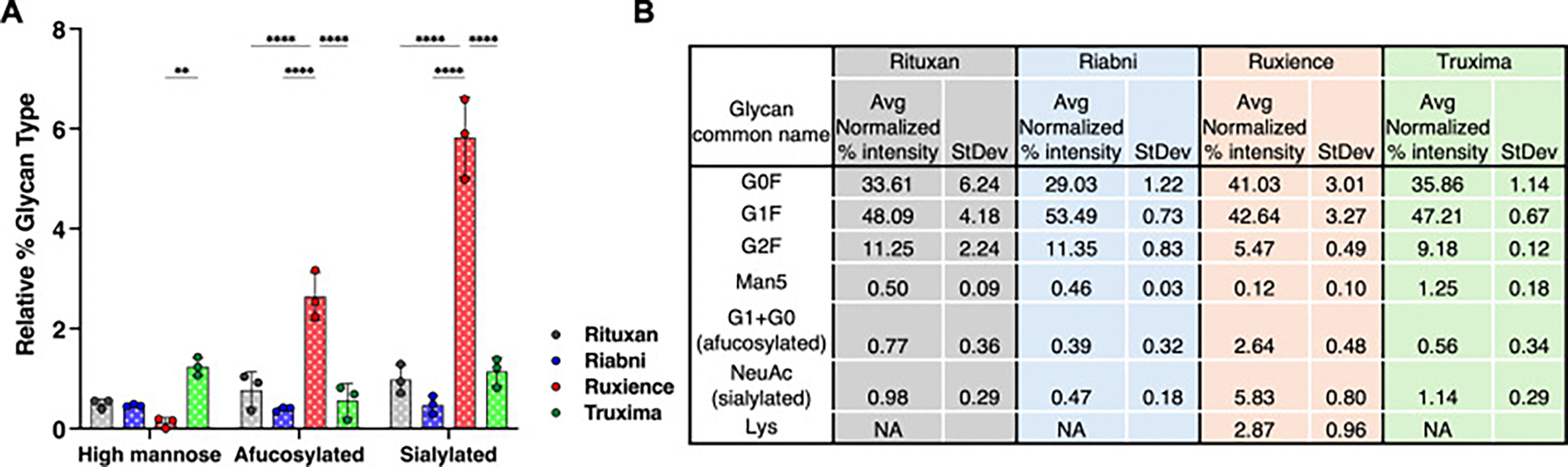
Relative percentage contribution of each glycan type by intact MS. (A) Relative percentage contribution of each glycan type in rituximab products, identified by intact MS. Detailed values are provided in Table (B). Lot *N* = 3; Statistical analyses were performed using Tukey’s multiple comparison test; *: *p* < 0.05; **: *p* < 0.01; ***: *p* < 0.001; ****: *p* < 0.0001. Note: Glycans appearing as pairs in the spectra were normalized to represent individual contributions.

Rituximab therapeutics are glycosylated at N301 on the HC. The deconvoluted spectra for the Ruxience (biosimilar) and Rituxan (reference) are depicted in Fig. 1b. Deconvoluted mass profiles of fully glycosylated revealed that the most abundant glycoforms were G0F/G0F, G0F/G1F, G1F/G1F (or G0F/G2F), and G1F/G2F with 4 pyroGlu formations and 2C-terminal Lys clips, as expected, exhibiting masses of 147,077 Da, 147239 Da, 147401 Da and 147,563 Da, respectively. These correspond to mass increments of +2887 Da (G0F/G0F), +3049 Da (G1F/G0F), +3211 Da (G1F/G1F or G0F/G2F) and + 3373 Da (G1F/G2F) compared to the deglycosylated sample. A Man5/Man5 peak was detected with relative intensities of 0.50 ± 0.09 %, 0.46 ± 0.03 %, 0.12 ± 0.10 %, and 1.25 ± 0.18 % in Rituxan, Riabni, Ruxience, and Truxima, respectively (Fig. 2 and Fig. S2). Afucosylated glycoforms (G1 + G0) were also observed, with Ruxience showing the highest relative abundance (2.64 ± 0.48 %) among the four products. Additionally, sialylated glycoforms with an addition mass of *N*-acetylneuraminic acid (NeuAc; +291.2 Da) were detected at a relative intensity of 0.98 %, 0.47 %, 5.83 %, and 1.14 % in Rituxan, Riabni, Ruxience, and Truxima, respectively. Notably, while most glycosylation profiles showed no C-terminal Lys (0 K), distinct glycosylation patterns were observed in Ruxience, which exhibited both 0 K and 1 K variants (Fig. 1b). These variants demonstrated an increase of 128.2 Da of G0F/G0F, G0F/G1F and G1F/G1F glycoforms at 2.87 ± 0.96 % of the total in Ruxience, while no such Lys variants were detected in the other rituximab product samples.

The rituximab samples were treated with peptide N-glycosidase F (PNGase F) to remove N-linked glycans, reducing the oligosaccharide complexity at the intact protein level. The theoretical mass of the deglycosylated rituximab was calculated to be 144,186.64 Da, assuming the presence of 4 pyroGlu modifications and 2C-terminal Lys clips. Deconvoluted mass profiles of PNGase F-treated rituximab products revealed a broad reference peak corresponding to masses of 144,192.9 ± 1.2 Da, 144194.5 ± 0.5 Da, 144194.8 ± 0.4 Da, and 144,194.4 ± 0.4 Da for Rituxan, Riabni, Ruxience, and Truxima (Table 1). These observed masses were between 5.1 Da and 8.6 Da higher than the theoretical MW. To investigate these peaks further, the deconvoluted spectra were refined using the intact MS Byos workflow, which revealed 2 distinct peaks corresponding to masses of 144,186.6 Da and 144,203.8 Da. These peaks were attributed to the reference form (+4pyroGlu, −2Lys) and a variant with 3 pyroGlu formations and 2C-terminal Lys clips, respectively. The observed masses of these two peaks were between 0.3 Da and 3.8 Da higher than the calculated theoretical masses. A significant difference was observed in the mass profiles of Ruxience, where an unclipped C-terminal Lys was detected. This lysine variant, which was also detectable in glycosylated MS spectra with low intensity, is highlighted in Fig. 1c within the sharpened deglycosylated spectra. The presence of the unclipped C-terminal Lys results in a mass shift of approximately +128.2 Da. In the deglycosylated samples, Ruxience exhibited the highest relative abundance of lysine variants, accounting for 11.71 ± 0.58 % of the total peak intensity, while Riabni showed 1.04 ± 0.56 %. In contrast, Rituxan and Truxima displayed lysine variant peaks of less than 0.5 % (Fig. S3a and Table 1). Also, the presence of the unclipped C-terminal Lys peaks was also observed in the unsharpened spectra (Fig. S3b), where Ruxience and Riabni exhibited intensities of 18.10 ± 0.81 % (1 K: 14.41 %, 2 K: 3.70 %) and 1.17 ± 1.17 % (1 K) of the total intensity, respectively.

### 3.2. Glycan mapping chromatograms

**Fig. 3.**
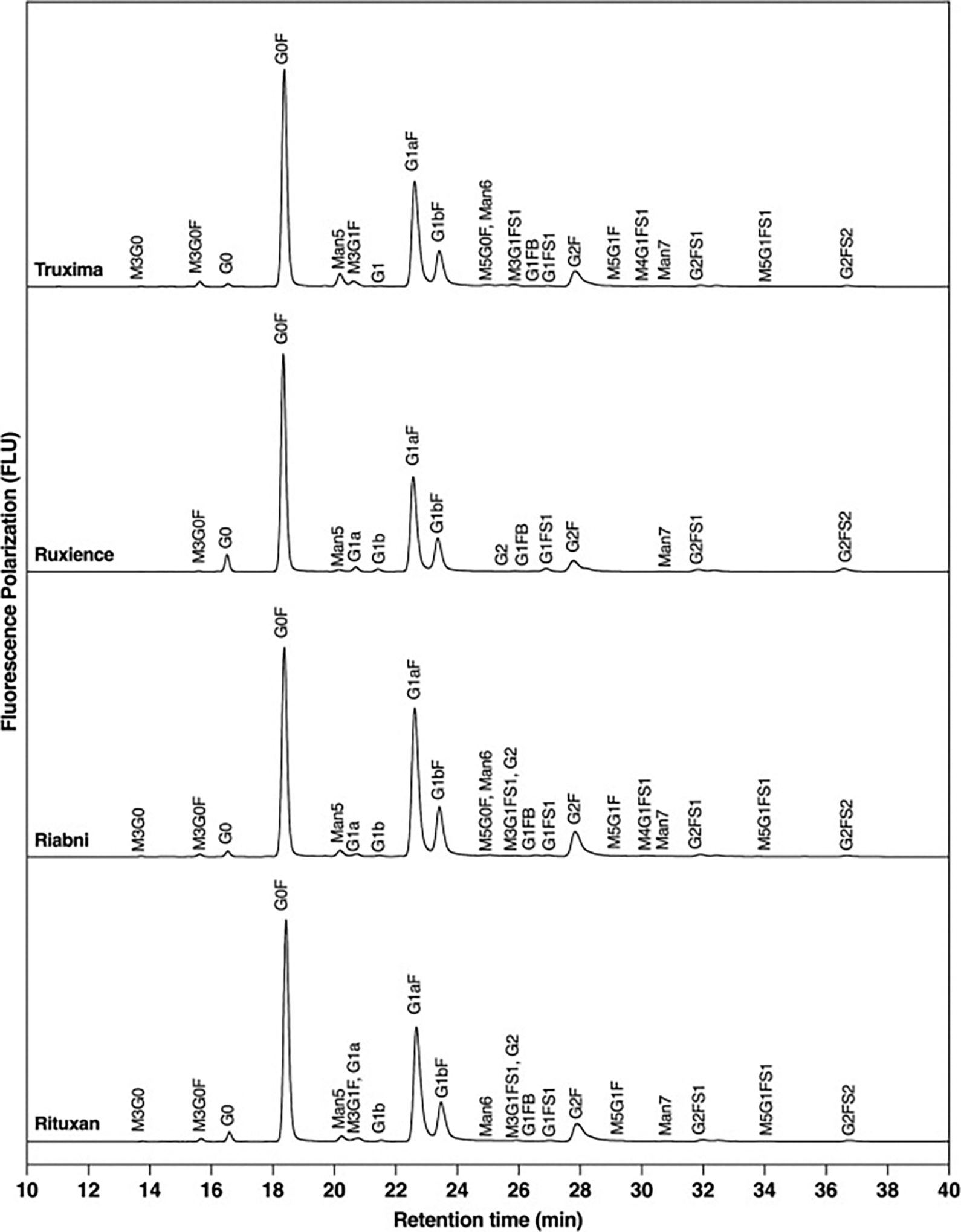
N-glycan profiles of rituximab products by fluorescence LC analysis. LC plots of released glycan which labeled with instant PC. Glycans were detected using fluorescence and identified using the Protein Metrics released glycan workflow. (Lot *N* = 3).

Glycosylation of the HC of rituximab is observed at the Fc region, specifically at position N301. An Instant PC kit exhibiting high signal intensity with high sensitivity and resolution, was used for glycan mapping. Fig. 3 depicts an Instant PC fluorescence chromatogram of the glycans from rituximab products, revealing a total of 22 different unique glycans. Rituxan, Riabni, and Truxima each contain 19, 20, and 20.67 unique glycans, respectively, while Ruxience contains only 13.33 unique glycans. Ruxience’s few unique glycans can be attributed to the absence of hybrid glycans compared to other rituximab antibodies.

**Fig. 4.**
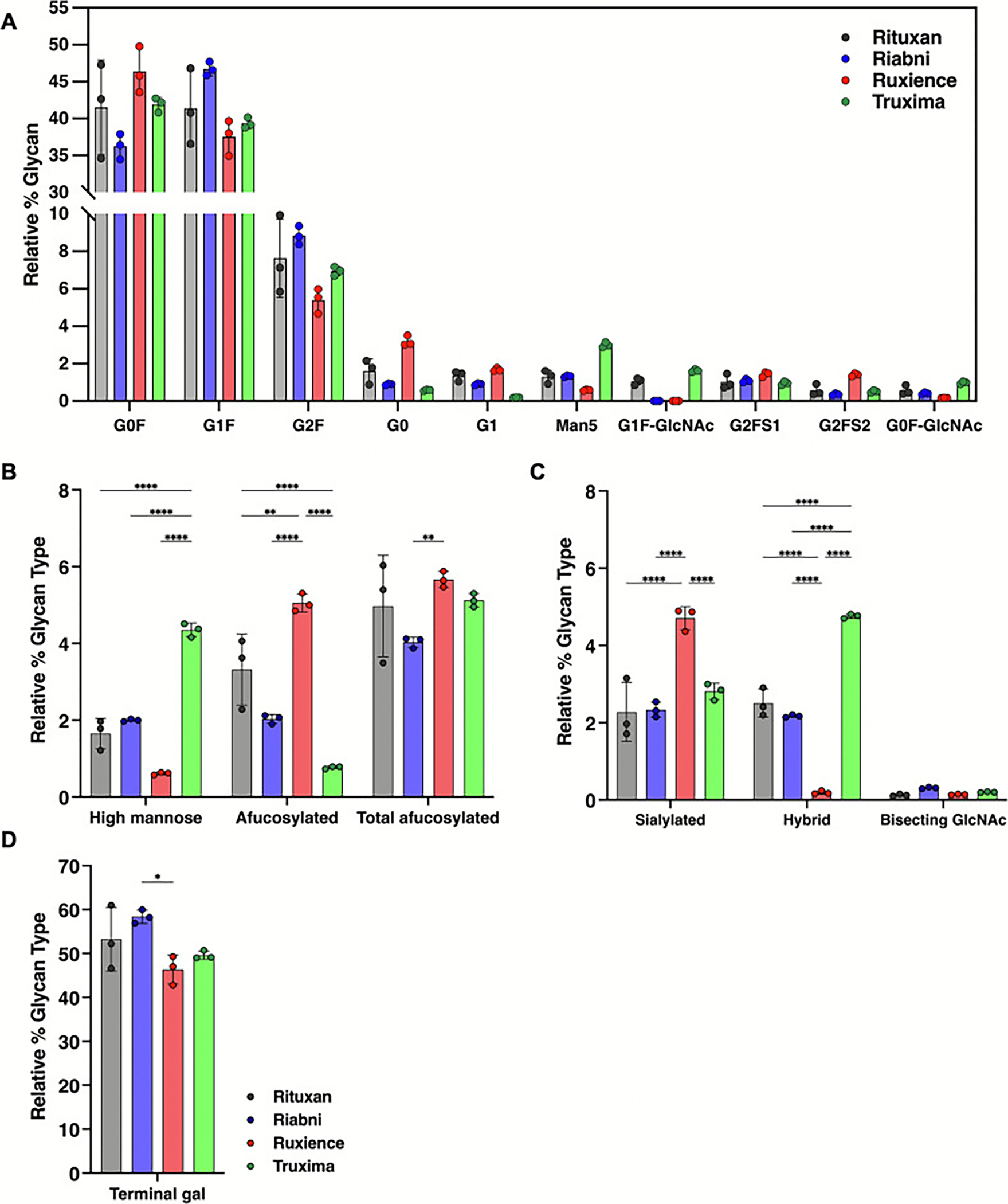
Relative percentage contribution of each glycan type by released glycan analysis (Instant-PC). (A) The top 10 glycans in Rituxan identified by LC-FLR-MS, categorized by glycan type: (B) percentage of mannosylated and afucosylated glycans, (C) percentage of sialylated, hybrid, and bisecting GlcNAc glycans, and (D) percentage of terminal galactosylated glycans. Glycans were identified using Protein Metrics. (Lot *N* = 3; error bars represent standard deviation. Statistical analyses were performed using Tukey’s multiple comparison test; *: *p* < 0.05; **: *p* < 0.01; ***: *p* < 0.001; ****: *p* < 0.0001.)

The main 3 identified glycans are complex fucosylated biantennary oligosaccharides, with 0 to 2 non-reducing galactoses (G0F, G1F and G2F). Other glycans observed include afucosylated biantennary glycans and high-mannose glycans (Fig. 4a and Table 2). The average normalized % areas for these major glycans were as follows: G0F was most abundant in Ruxience (46.38 %), while Riabni (36.25 %) showed lower values. G1F exhibited the highest abundance in Riabni (46.70 %), followed by Rituxan (41.40 %), Truxima (39.36 %), and Ruxience (37.52 %). G2F showed the highest value in Riabni (8.83 %), followed by Rituxan (7.63 %), Truxima (6.94 %), and Ruxience (5.39 %). In Rituxan, lot1 contain more G1F (46.89 %) than G0F (34.62 %), while lot2 and lot3 exhibited G0F (42.63 % and 47.28 %) outnumbering G1F (40.77 % and 36.55 %). This resulted in almost equal average amounts of G0F and G1F across the lots. In Riabni, the G1F > G0F > G2F distribution was consistent across all lots, whereas in Ruxience and Truxima, G0F > G1F > G2F was the predominant pattern. The fourth most abundant glycan differed between the rituximab products: G0 was the fourth most abundant glycan in Rituxan and Ruxience, whereas Man5 occupied this position in Riabni and Truxima. Ruxience exhibited a 2-fold higher level of G0 (3.19 %) compared to Rituxan (1.61 %), while Truxima showed 2.7-fold lower G0 levels (0.58 %). In contrast, Truxima had a 2.3-fold higher abundance of Man5 (2.99 %) compared to Rituxan (1.30 %) and Riabni (1.33 %), while Ruxience had a 2.2-fold lower level (0.59 %).

Glycans we were particularly interested in monitoring were afucosylated, mannosylated, sialylated, terminal galactosylated, hybrid and bisecting GlcNAc. The relative % contribution of these glycan groups were calculated from all integrated peaks. Terminal galactosylated glycans, including G1, G1F, G1FB, G1F-GN, M5G1F, G2, G2F, and G2FS1, were most abundant in Riabni (58.36 ± 1.57 %), following by Rituxan (53.26 ± 7.29 %), Truxima (49.57 ± 0.96 %), and Ruxience (46.35 ± 3.28 %) (Fig. 4d). Notably, α-1,3-galactose (α-gal) was not detected in the released glycan analysis. Among the different glycan types, Truxima exhibited the highest relative percentage of high-mannose (4.35 ± 0.18 %) and hybrid (4.76 ± 0.06 %) glycans (Fig. 4b and c). In contrast, Ruxience showed the highest relative abundance of afucosylated (G0 + G1 + G2) (5.05 ± 0.23 %) and sialylated (4.71 ± 0.30 %) glycans. Bisecting GlcNAc glycan, G1FB (G1F + GN), was detected in small amounts across all rituximab samples, but no statistically significant differences were observed in their levels.

### 3.3. Peptide mapping

**Fig. 5.**
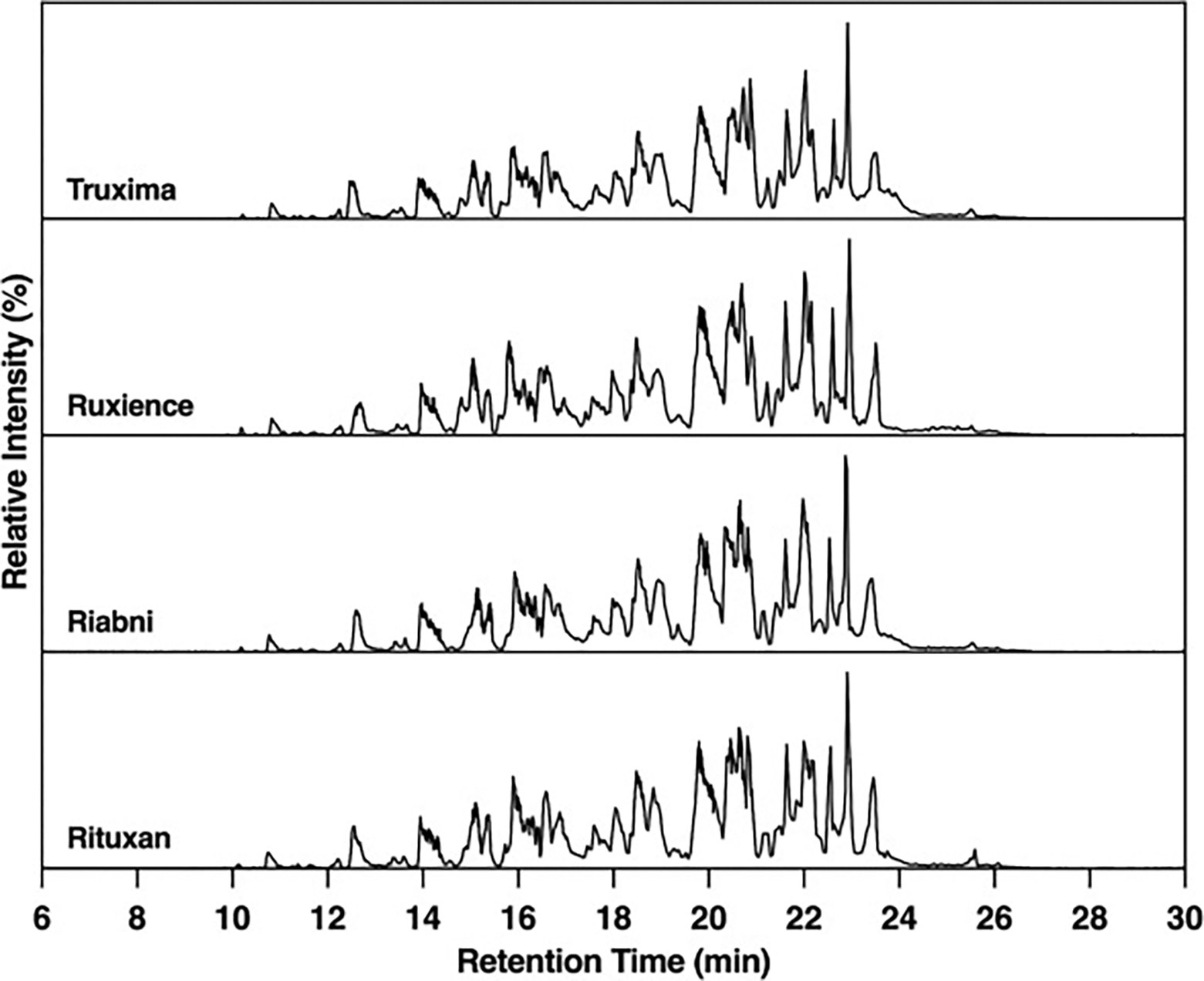
Total ion chromatogram (TIC) of tandem MS analysis of rituximab products obtained in peptide mapping experiments. Approximately 500 ng of each product was loaded on the column.

To further investigate differences across the reference and biosimilar rituximab products, we employed LC-MS/MS as an orthogonal method for peptide mapping. The TICs generated post digestion were illustrated in Fig. 5. Both reference and biosimilars had identical sequence with 100 % (213 out of 213) coverage for LC and 97.12 % (438 out of 451) coverage for HC. Peptide mapping by LC/MS/MS was used to evaluate the N-glycosylation profile and the extent of the N-terminal PyroGlu formation, and C-terminal lysine clipping, and unravel the modification hotspots including oxidation and deamidation.

#### 3.3.1. N-glycosylation

N-glycosylation was observed at the N301 residue within the heavy chain peptides sequences (1) TKPREEQYN^301^STYR (2) EEQYN^301^STYR (3) EEQYN^301^STYRVVSVLTVLHQDWLNGK for rituximab. Nearly the entire peptide population was glycosylated, with the intact deglycosylated peptide representing 0.84 ± 0.26 %, 1.20 ± 0.47 %, 0.63 ± 0.25 %, and 1.93 ± 0.68 % of the total peptide content in Rituxan, Riabni, Ruxience, and Truxima, respectively. A total of 45 distinct glycans were detected with ≥0.01 % relative abundance, with 43, 35, 35, and 39 different glycans identified in Rituxan, Riabni, Ruxience and Truxima, respectively (Table 3).

**Fig. 6.**
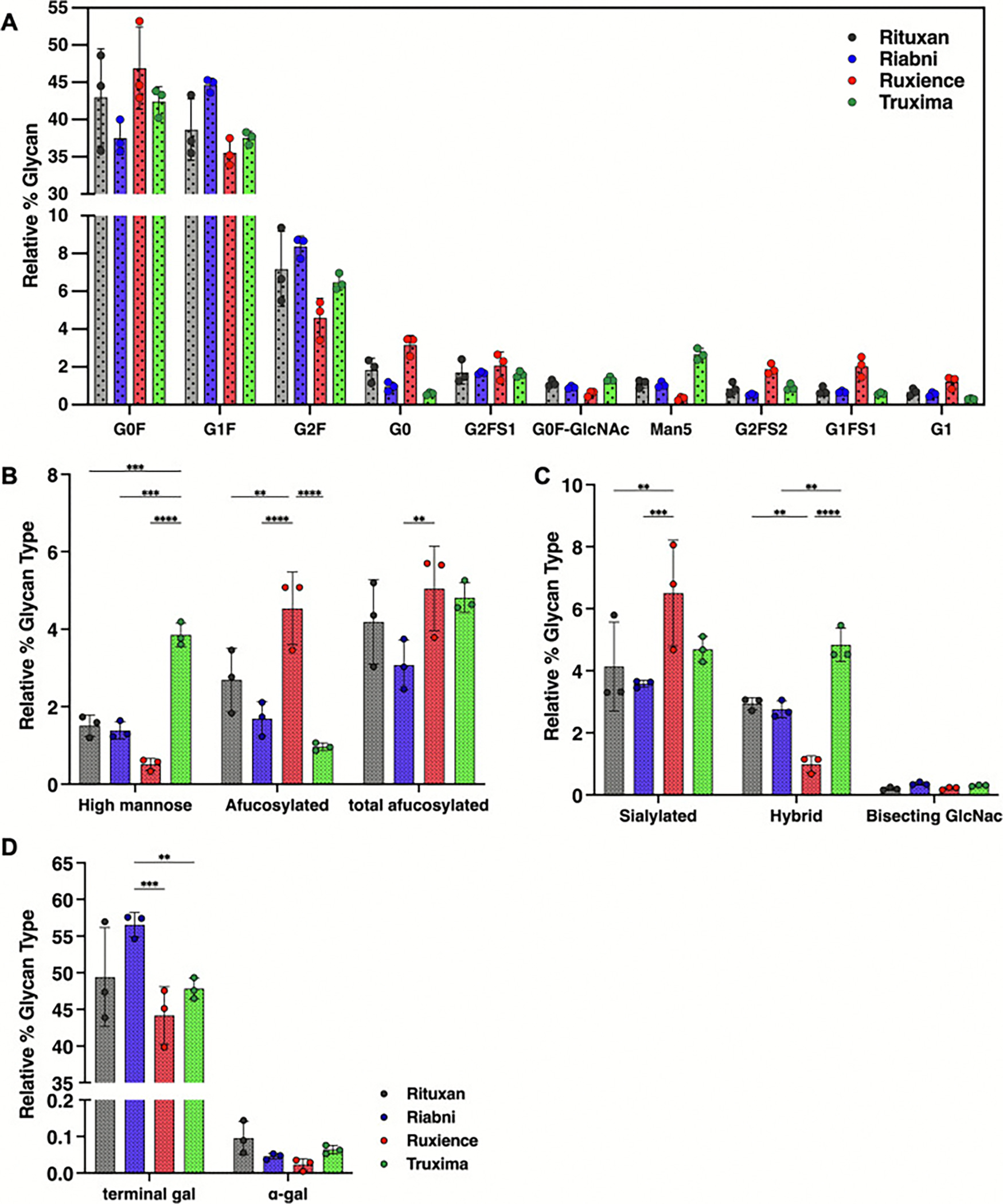
Relative percentage contribution of each glycan type by peptide mapping analysis (LC-MS/MS). (A) The top 10 glycans in Rituxan identified by LC-MS/MS, categorized by glycan type: (B) percentage of mannosylated and afucosylated glycans, (C) percentage of sialylated, hybrid, and bisecting GlcNAc glycans, and (D) percentage of terminal galactosylated glycans. Glycans were identified using Protein Metrics. (Lot *N* = 3; error bars represent standard deviation. Statistical analyses were performed using Tukey’s multiple comparison test; *: *p* < 0.05; **: *p* < 0.01; ***: *p* < 0.001; ****: *p* < 0.0001).

Fig. 6a depicts the relative % N-glycans of four different rituximab products. Among these, G0F showed the following average normalized XIC % areas: 42.97 % (Rituxan), 37.50 % (Riabni), 46.90 % (Ruxience) and 42.43 % (Truxima). For G1F, the values were 38.63 % (Rituxan), 44.63 % (Riabni), 35.53 % (Ruxience) and 37.53 % (Truxima), while for G2F, the values were 7.17 % (Rituxan), 8.36 % (Riabni), 4.59 % (Ruxience) and 6.47 % (Truxima). In line with the result of the released glycan assay, in Rituxan, the average amount of G0F was higher than G1F, although a slight lot-to-lot variation was observed. The G1F > G0F > G2F distribution pattern was consistent across all the lots of Riabni, while G0F > G1F > G2F was the dominant distribution observed in Ruxience and Truxima. The fourth most abundant glycans varied across rituximab products. In Rituxan and Ruxience, G0 was predominant, while in Riabni and Truxima, G2FS1 and Man 5 were the fourth highest, respectively. Ruxience exhibited a 1.7-fold higher quantity of G0 compared to Rituxan (3.15 % vs 1.83 %), while Truxima contained 3.1-fold less G0 than Rituxan (0.58 % vs 1.83 %), which agrees with the released glycan data. G2FS1 (NeuAc), although the fourth highest glycan in Riabni (1.67 %), was present at similar levels in all rituximab products, with Ruxience showing a slightly higher proportion (2.07 %). Conversely, Man5 was quantified to 2.3-fold higher in Truxima (2.66 %) than Rituxan and Riabni (1.12 % and 1.05 %, respectively), while Ruxience exhibited a 3-fold lower amount (0.33 %) compared to Rituxan and Riabni.

Peptide mapping was able to reveal additional unique glycans, such as those containing N-glycolylneuraminic acid (NeuGc), a hydroxylated derivative of NeuAc, (e.g., G2F + NeuGc and G2F + NeuAc+NeuGc), and those containing α−1,3-galactose (α-gal), known as non-human N-glycan epitopes (e.g., G2F + Gal1 and G2F + Gal1 + NeuAc). NeuGc represented 0.05 %, 0.21 %, and 0.01 % of the total glycans in Rituxan, Ruxience, and Truxima respectively. α-Gal constituted 0.10 %, 0.05 %, 0.02 %, and 0.06 % of the total glycans in Rituxan, Riabni, Ruxience, and Truxima, respectively (Fig. 6d).

We also categorized the detected glycans into several types – afucosylated, mannosylated, sialylated, terminal galactosylated, hybrid and bisecting GlcNAc glycans (Fig. 6b, c, and d). These findings were consistent with the results from released glycan analysis. Terminal galactosylated glycans represented 56.53 ± 1.66 % (Riabni), 49.39 ± 6.77 % (Rituxan), 47.80 ± 1.43 % (Truxima) and 44.18 ± 3.93 % (Ruxience). Truxima showed a statistically significant higher relative % contribution of high-mannosylated glycans (3.85 ± 0.30 %) and hybrid glycans (4.84 ± 0.54 %), while Ruxience had the highest relative percentage of afucosylated (G0 + G1 + G2) glycans (4.54 ± 0.94 %) and sialylated glycans (6.50 ± 1.71 %) compared to the other products. The total afucosylated glycans (high-mannose+G0 + G1 + G2) were 5.05 ± 1.09 % for Ruxience, 4.82 ± 0.38 % for Truxima, 4.19 ± 1.09 % for Rituxan, and 3.08 ± 0.65 % for Riabni.

#### 3.3.2. Amino acid modification

Table 4 listed the tryptic peptides that contain the modification spots. Aforementioned, rituximab possesses an N-terminal Q1 on both HC and LC, forming an N-terminal pyroGlu, and a C-terminal lysine (K451) in the HC which is susceptible to be clipped. The average normalized XIC % areas for the modified peptides are shown. All rituximab products exhibited over 98 % pyroGlu formation at the HC N-terminus, while the LC showed greater variability in this modification. Ruxience, for instance, demonstrated a significantly lower level of pyroGlu formation on the LC (74.13 %) compared to other rituximab products. Furthermore, a markedly higher abundance of unclipped C-terminal Lys was observed in Ruxience (8.49 %) relative to the other rituximab products.

Oxidation of methionine and tryptophan occurs in mAbs during cell culture, purification, formulation, and storage processes, which potentially results in decreased bioactivity and stability of mAbs. Methionine sulfoxide results from Met oxidation and corresponds to a + 15.9949 Da increase. The modification can also include the elimination of methyl sulfonic acid (CH_3_SOH), leading to dethiomethylation (−48.0034 Da). In addition, tryptophan can undergo oxidation (+15.9949 Da) or dioxidation (+31.9898 Da).

**Fig. 7.**
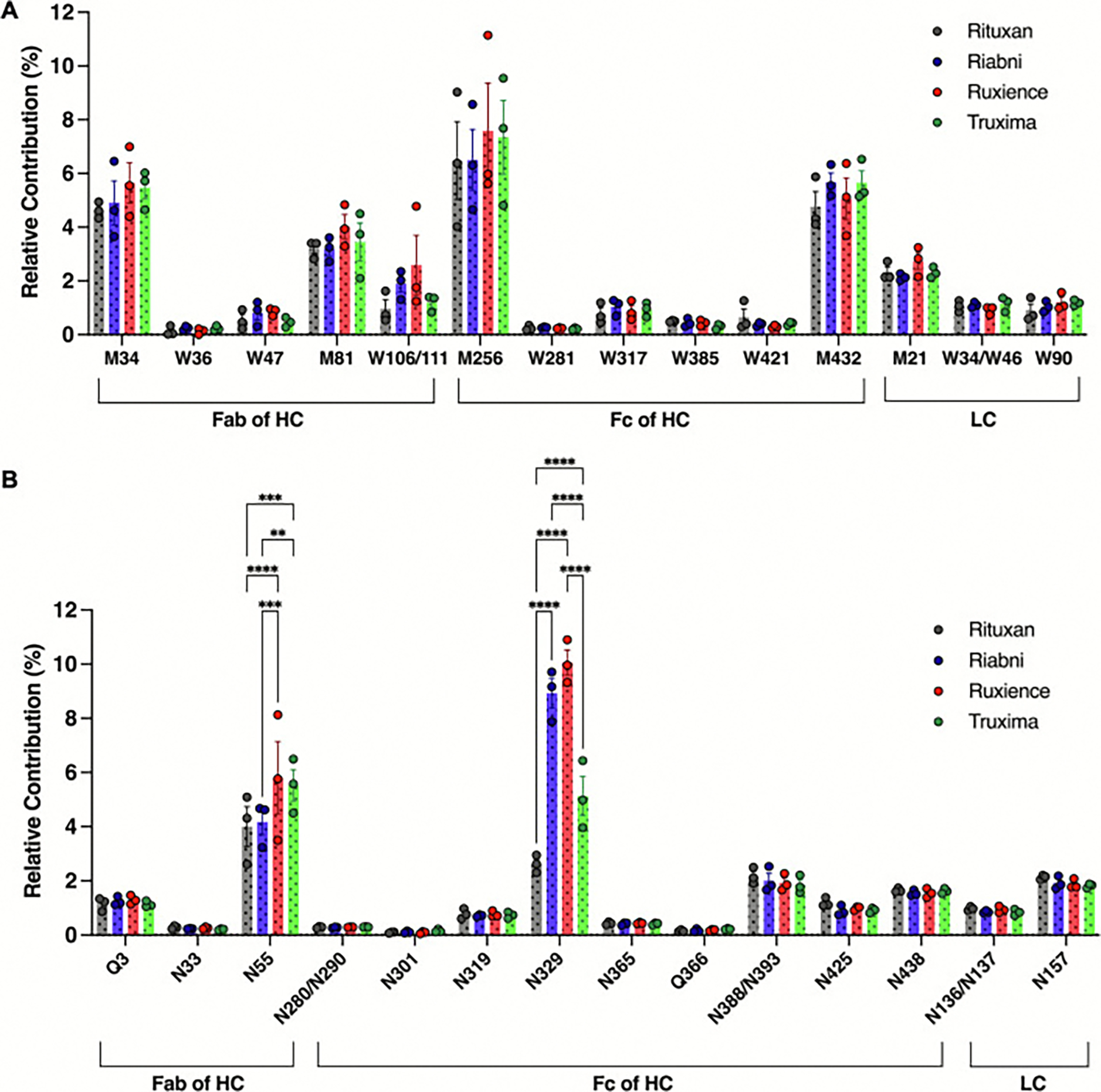
LC-MS/MS analysis of tryptically digested rituximab products. (A) Total contribution of oxidation, dioxidation, and dethiomethylation at Trp (W) or Met (M) residues, relative to the XIC AUC sum of all identified peptides. (B) Total contribution of succinimidation and deamidation at Asn (N) or Gln (Q) residues, relative to the XIC AUC sum of all identified peptides. These modifications were detected in rituximab samples by LC-MS/MS through peptide mapping. HC: Heavy Chain; LC: Light Chain. (Lot *N* = 3; error bars represent standard deviation. Statistical analyses were performed using Tukey’s multiple comparison test; *: *p* < 0.05; **: *p* < 0.01; ***: *p* < 0.001; ****: *p* < 0.0001.)

In rituximab, a total of sixteen possible oxidation sites have been identified, including five Met and eleven Trp residues. Fig. 7a illustrates the relative % of oxidized residues across different samples. Among these, M34 and M81in the Fab region of HC, as well as M256 and M432 in Fc region of HC exhibited comparatively high oxidation levels, although no statistical differences were observed across the different rituximab products.

Notably, two highly conserved Met residues in the Fc fragment of human IgG1—M256 and M432, located at the C_H_2-C_H_3 interface—demonstrated particularly high oxidation levels. M256 in the DTLM^256^ISR tryptic peptide was found to be especially prone to oxidation [26]. As shown in Fig. S4, representative peptide DTLMISR and its oxidized forms were successfully extracted from the total ion chromatogram and confirmed by accurate molecular ions and MS/MS fragment ions. In the chromatogram, the peak corresponding to the dethiomethylated M256 peptides eluted 3 min earlier, while the oxidized M256 peptides eluted 2 min earlier compared to the wild-type (WT) peak under reversed-phase conditions. MS/MS spectra of the DTLMISR peptide containing either dethiomethylated or oxidized M256 revealed diagnostic ions for those modifications (Fig. S5 a–c). For rituximab products, dethiomethylation of M256 was observed at levels around 2–3 %, while oxidation occurred at approximately 4.5 %. Similarly, M432 was found to exhibit 1.0–1.4 % dethiomethylation and 3.5–4.5 % oxidation in three tryptic peptides. These residues correspond to M255 and M431 in NIST mAb, with 4.94 % and 3.20 % modification, including dethiomethylation and oxidation, which are comparable to rituximab product samples.

For all rituximab products, the combined oxidation and dethiomethylation of M34 and M81 in the variable heavy (VH) region averaged 4.63–5.65 %, and 3.19–4.02 %, respectively. The critical residues involved in rituximab-CD20 recognition, W106 and W111 in the CDR H3 region, are adjacent to M81[27]. Due to the proximity of W106 and W111, it was not possible to distinguish which of the tryptophan residues were oxidized. The tryptic peptide sequence detected in this region was STYYGGDW^106^YFNVW^111^GAGTTVTVSAASTK, where oxidation peptides were detected at the levels between 0.96 % and 2.59 % in each rituximab products, with no detectable dioxidation modifications. Additionally, other residues showing oxidation modifications greater than 1 % include M21, W34/46 and W90 in the variable light (VL) region. M21 and W90 in the LC, located at CDR L1 and L3, respectively, exhibited approximately 2 % modifications, with no significant differences observed between different rituximab products. W34/W46 displayed oxidation levels around 1 % (with dioxidation levels between 0.36 % and 0.48 %, and oxidation levels between 0.52 % and 0.67 %).

Deamidation of Asn and Gln residues in biological pharmaceuticals is a major cause of chemical degradation. Deamidation caused a mass increase of 0.9840 Da. The XIC revealed that the peptides appeared slightly shifted relative to the WT peptides (Fig. S6). For deamidation to occur, Asn residues first form a succinimide intermediate by loss of an ammonia (−17.0265 Da) and subsequently undergo hydrolysis, resulting in a deamidation with a 0.9840 Da increase.

Peptide mapping unraveled eighteen potential degradation hotspots, comprising two Gln and sixteen Asn residues. Among these, two Gln (Q3 and Q366 in the HC) and seven Asn residues (N55, N301, N365, N425 in the HC and N136/N137, N157 in the LC) were found to undergo deamidation at levels not exceeding 1.5 % at each residue. Fig. 7b shows the relative % of the sum of deamidated and succinimidated residues.

In particular, N55 in the CDR H2 region of the Fab domain of the HC and N329 in the C_H_2 of the Fc domain of the HC exhibited significantly higher levels of degradation (here referring to deamidation and succinimidation), with statistical differences observed across the different rituximab products. These residues are highly conserved among IgG1 mAbs. The representative peptide GLEWIGAIYPGNGDTSYN^55^QK and its succinimidated and deamidated forms were successfully detected in the total ion chromatogram and MS/MS fragment ions (Fig. S6). MS/MS spectra of the peptides containing either succinimidated or deamidated N55 revealed diagnostic ions for those modifications (Fig. S7a–c). Quantification of succinimidation levels showed that Ruxience (5.12 ± 2.11 %) and Truxima (4.88 ± 1.05 %) exhibited statistically higher levels on N55 compared to Rituxan (3.77 ± 1.28 %) and Riabni (3.75 ± 0.65 %). Similarly, for deamidation, the average deamidation levels were observed 0.23 %, 0.42 %, 0.68 %, and 0.64 % for Rituxan, Riabni, Ruxience, and Truxima, respectively. In case of N329, Ruxience (10.06 ± 0.79 %) and Riabni (8.92 ± 0.94 %) displayed significantly higher levels of succinimidation compared to Rituxan (2.62 ± 0.33 %) and Truxima (5.13 ± 1.20 %), with no deamidation detected. NIST mAb’s HC N328, which shares the same tryptic peptide sequence as rituximab’s N329, contained 1.44 % succinimide intermediate without deamidation.

Except for these residues, the degradation patterns observed across all Rituximab samples exhibited similar trends, with comparable levels of succinimidation and deamidation at the identified hotspots in the different mAbs. N388 and N393 residues in the conserved C_H_3 region of the HC were found on the same tryptic peptide (GFYPSDIAVEWESN^388^GQPEN^393^NYK). In all rituximab samples, approximately 2 % succinimide formation was observed for this peptide, without any deamidation detected. As shown in Fig. 7b, N425 and N438 in the conserved Fc region were observed to undergo degradation, with N425 showing deamidation and succinimidation levels ranging from 0.24 % to 0.37 % for deamidation and 0.58 % to 0.83 % for succinimidation across all rituximab samples. In contrast, N438 showed only succinimidation, with degradation level of approximately 1.5 %. Finally, N157 in the C_L_ region of the LC exhibited deamidation levels between 0.65 % and 0.87 %, and succinimidation levels ranging from 1.17 % to 1.26 % across the rituximab products.

### 3.4. Biological activities

**Fig. 8.**
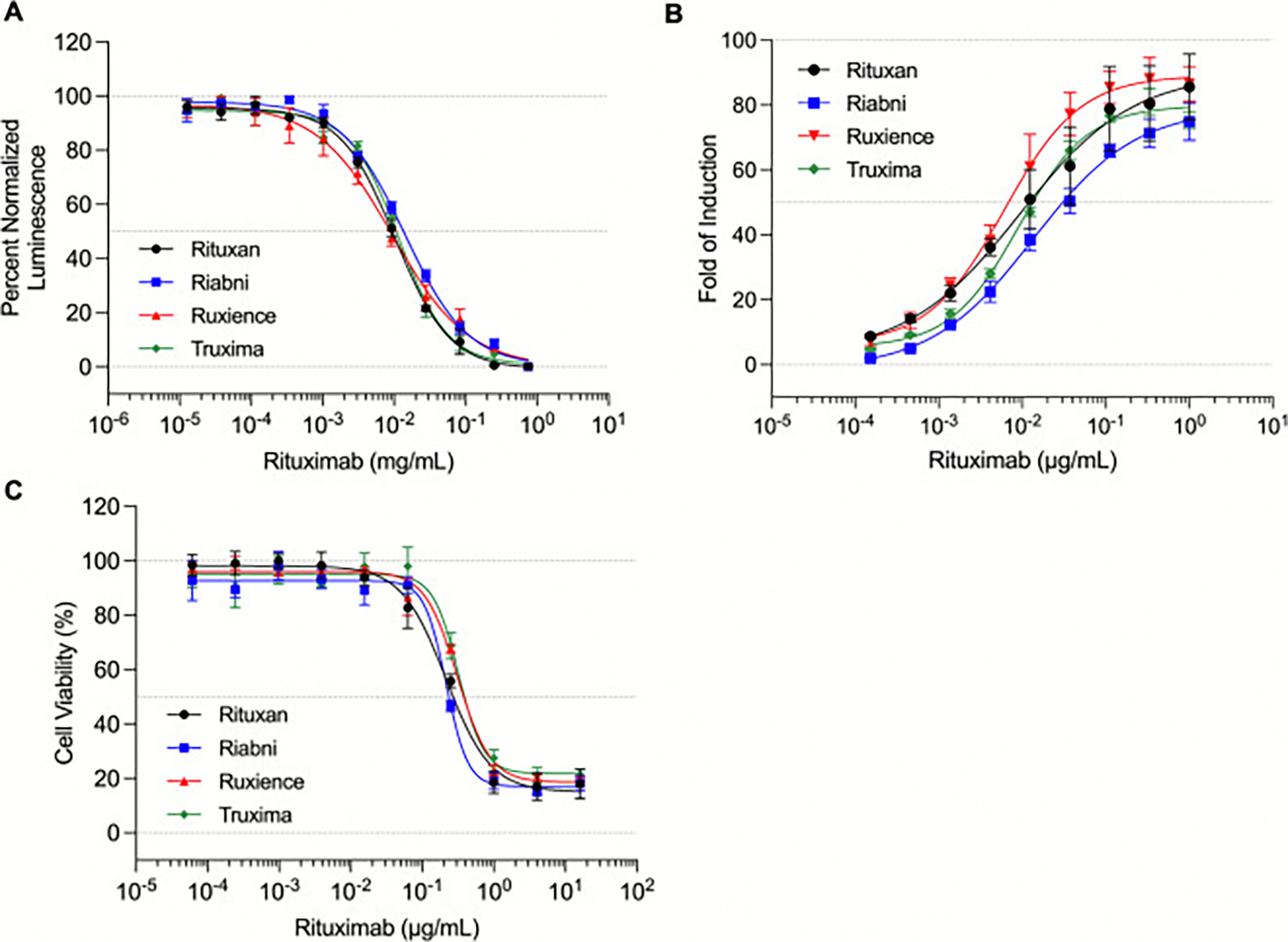
Comparison of the biological activities of rituximab products. (A) FcRγIIIa binding affinity curves show that Ruxience exhibits slightly higher binding activity compared with other products. Assay was performed using the Lumit FcRγIIIa V158 variant. (B) ADCC Reporter bioassay results confirm this trend, with Ruxience demonstrating the lowest EC_50_ value. The assay was performed using Wil2-S target cells and Jurkat effector cells expressing the high-affinity V158 variant. Fold of induction was calculated by dividing RLU (induced-background) by the RLU (no antibody control - background). (C) CDC assay results show comparable complement-mediated cytotoxicity among all samples. Wil2-S target cells were treated with rituximab samples and 10 % NHS. Data represent mean ± standard deviation of technical triplicates (*N* = 3); dose response curve was fitted with a 4-parameter model using GraphPad Prism^®^ software.

To compare the ADCC activity among rituximab products, FcRγIIIa binding affinity and ADCC reporter assays were performed. The ADCC reporter assay employed Wil2-S target cells and a Jurkat effector cells expressing the high-affinity FcRγIIIa V158 variant, while FcRγIIIa binding was measured using the Lumit FcRγIIIa (V158 variant) immunoassay. As shown in Table 5, Fig. 8a and b, both assays confirmed that all four products exhibited overall comparable FcRγIIIa binding affinities and ADCC potencies, with slight quantitative differences among biosimilars. Ruxience showed the highest FcRγIIIa binding affinity (IC_50_ = 9.51 μg/mL) and ADCC activity (EC_50_ = 6.06 ng/mL), whereas Riabni exhibited the lowest affinity (IC_50_ of 14.08 μg/mL) and ADCC activity (EC_50_ = 13.58 ng/mL) among rituximab products (Table 5). Additionally, to assess the effect of N-glycosylation ADCC, deglycosylated rituximab samples were also analyzed. After deglycosylation, a significant reduction in FcRγIIIa binding affinity (Fig. S8) and abolished ADCC activity (Fig. S9) were observed for all rituximab products.

CDC activity was also evaluated using Wil2-S target cells and NHS as a source of complement. As shown in Fig. 8c, all rituximab products exhibited comparable CDC potency. The EC_50_ values were as follow: Rituxan (reference), 0.214 μg/mL; Riabni, 0.215 μg/mg; Ruxience, 0.310 μg/mL; Truxima, 0.321 μg/mL. For some samples, reliable estimation of the 95 % confidence interval was not possible due to limited curve fitting or high variability in the CDC response. Therefore, only EC_50_ values are reported. These results indicate that all tested rituximab products exhibited comparable CDC activity within the expected variability range of the assay. To confirm sample integrity and ensure comparability of biological activity results, SEC-UPLC was performed prior to functional assays. In all rituximab products, no detectable aggregation or fragmentation was found (Fig. S10).

## 4. Discussion

The full characterization of the four different FDA-approved rituximab products were compared using various advanced MS-based analysis technologies. All analytical workflows were conducted under identical experimental conditions, allowing direct comparison across multiple orthogonal MS-based datasets and minimizing potential variability arising from sample handling or instrumentation. We successfully identified the full amino acid sequence and quantified the PTMs. Employing different quantification techniques—relative intensity for intact MS, LC-normalized area% for released glycan assay, and XIC area summation for peptide mapping—the results from all three methods were highly consistent. Validation of the N-glycosylation profiles for selected products revealed a good concordance across different analytical methods, further supporting the reliability of the observed data.

Minor discrepancies were observed only for ultra-low abundance glycoforms, which were detectable by MS/MS but not resolved in released glycan analysis due to sensitivity and peak separation limits. In particular, the trace levels (0.02–0.10 %) of α-Gal–containing glycans were identified in LC–MS/MS peptide mapping data, where site-specific fragmentation enables identification of low-abundance species. In contrast, released glycan profiling is less sensitive to ultra-trace species. In the case of NeuGc, MS/MS allowed detection and quantification based on their *m*/*z* values, whereas in released glycan profiling, such glycans likely co-eluted with structurally similar NeuAc glycans and were masked by the predominant NeuAc peaks. A minor NeuGc contribution was observed in the retention region of the major NeuAc peak, but due to co-elution and the much higher NeuAc signal intensity, the peak was assigned as NeuAc in the released glycan profile.

For biosimilar approval, structural differences between reference and the candidate biosimilar were thoroughly evaluated. While the structural differences between reference and biosimilar were expected to be minimal because they were all shown to be highly similar to their reference product, differences among biosimilars could be more pronounced. This was particularly evident in the comparative N-glycan analysis, a critical feature of therapeutic antibodies that influences biological functions [28]. The analysis showed that Ruxience and Truxima exhibited greater differences from each other than from Rituxan. This was especially apparent in the high-mannosylated glycan profile and hybrid glycans, where Truxima showed the highest abundance percentage, whereas the lowest amounts were found in Ruxience. Additionally, Ruxience exhibited the highest level of afucosylated glycans, while Truxima had the lowest level among the rituximab products tested. These trends of glycan profiles were identified in both LC-FLR and tandem MS methods. On the other hand, Riabni exhibited the most similar composition to the reference product, Rituxan.

According to the biosimilar regulatory documents [29,30], glycan analysis was considered as moderate or mild in terms of its impact on ADCC and CDC activity, and capability of impact on immunogenicity. In our study, the removal of N-linked glycans in the Fc region caused a major decrease in its ability to bind to FcRγIIIa on immune cells (Fig. S8), which in turn prevents the ADCC immune response (Fig. S9). This confirmed that glycosylation is essential for the rituximab’s Fc-mediated effector function.

However, the differences on glycan composition were not directly translated into the significant differences on ADCC, as each glycan type affects ADCC differently. For example, afucosylated (G0 + G1 + G2) glycans, which are associated with enhanced ADCC due to increased FcγRIIIa binding affinity [28], were observed at varying levels across products in the following order: Ruxience > Rituxan > Riabni ≥ Truxima. In contrast, high-mannosylated glycoforms, also linked to enhance ADCC [31], were most abundant in Truxima and least in Ruxience. Terminal galactosylation, which may have a modest effect on ADCC [32], followed the order: Riabni ≥ Rituxan > Truxima ≥ Ruxience. Sialylated glycans, generally associated with reduced ADCC potential due to anti-inflammatory properties [33,34], were most prominent in Ruxience. While these glycan profiles reflect known associations with ADCC activity, no conclusions about relative clinical potency should be drawn, as all products included in this study met the regulatory standards for approval. Rather, these findings illustrate how analytical glycoform profiling can highlight subtle structural differences among approved rituximab products.

We investigated the ADCC activity using commercialized PBMC effector cells between the biosimilars and reference rituximab.

Although the glycan compositions of Ruxience and Truxima showed the biggest difference, the potency calculated by our ADCC assay did not show significant variation between them, as their EC_50_ values overlapped within 95 % confidence interval. As we could see in the structural differences, the differences on potency between reference and biosimilar were minimal, while Riabni (EC_50_ 13.58 ng/mL) and Ruxience (EC_50_ 6.06 ng/mL) showed statistically significant differences. The order of ADCC potency was aligned with the total afucosylated glycan abundances (high-mannose+G0 + G1 + G2), with the ranking being: Ruxience ≥ Truxima ≥ Rituxan ≥ Riabni, demonstrating a statistically significant difference between Ruxience and Riabni. These results suggest that despite the balanced glycan effects on ADCC, the total afucosylated glycan abundance correlates strongly with in vitro ADCC. And this trend is also observed in FcγRIIIa binding. Nonetheless, while the observed differences in ADCC potency and FcγRIIIa binding among rituximab products generally correlated with the total afucosylated glycans, definitive attribution of functional differences to individual glycoforms is limited by the complexity of glycan interplay and by the experimental resolution of the dataset.

We also examined CDC activity to evaluate another Fc-mediated effector function of rituximab (Fig. 8c). The trends observed in our study were generally consistent with the levels of terminal galactosylation, which is known to affect C1q binding and thereby modulate CDC response [35]. Riabni and Rituxan, which exhibited higher galactosylation, showed slightly stronger CDC potency (EC_50_ = 0.215 μg/mL and 0.214 μg/mL, respectively, compared to Truxima and Ruxience, which had lower terminal galactosylation. Moreover, previous reports indicate that both sialylation and high-mannose content can reduce CDC activity due to impaired C1q interaction [31,36]. Consistent with this, Ruxience, with the highest sialylated glycan, and Truxima, with the most abundant high-mannose glycans, showed lower CDC potency. These findings suggest that excessive sialylation or high-mannose glycan may blunt CDC responses.

It is also important to note that CDC assays generally show greater experimental variability than the standardized ADCC report assay that used in this study. Complement activity depends on the donor serum used as the complement source and may vary between experiments, although we used commercial NHS to reduce such variability. In addition, the complement sensitivity of the target cell, Wil2-S, is not constant, which can contribute to assay-to-assay variation. Therefore, although the overall CDC trends aligned with glycan composition, the magnitude of these differences should be interpreted with caution.

We have previously demonstrated that reference, Rituxan, exhibited 98.5 ± 0.1 % formation of N-terminal pyroGlu for the HC and 81.2 ± 6.4 % for the LC [37], which is consistent with our finding where we found 99.03 ± 0.47 % and 86.90 ± 0.85 %, respectively. This confirms that peptide mapping is a robust analytical method for comparing pyroGlu formation. Among the rituximab products analyzed, Ruxience exhibited the highest observed variability in N- and C- terminal sequences of both HC and LC. This was primarily driven by differences in the extent of N-terminal pyroGlu formation in the LC (74.13 ± 4.92 %) and C-termina Lys clip modifications in the HC (91.5 ± 4.85 %), both of which are common PTMs that do not impact clinical performance. All Rituximab samples showed a distinct feature of a higher remaining ratio of N-terminal Gln of the LC compared to that of the HC, resulting in the main peak of the intact MS spectra of deglycosylated mAbs being divided into two peaks (Fig. 1c). The intact MS spectrum of deglycosylated NIST mAb only contained one major reference peak (Fig. S11), since NIST mAb only contains N-terminal Gln on the HC, where pyroGlu-formed tryptic peptide (qVTLR) was detected 99.93 % in the peptide mapping. Since the mass difference due to pyroGlu formation is 17 Da, quantifying the pyroGlu formation from the intact MS was difficult, whereas the extent of Lys clipping can be roughly quantified by the intact MS. For example, the high remaining C-terminal unclipped Lys of Ruxience showed 18.66 ± 0.87 % in the unshapen deglycosylated intact MS spectrum (Table 1). Considering that the native mAb consists of 2 LCs and 2 HCs, Ruxience containing one or two Lys unclipped HC would be 16.12 ± 8.74 % calculated by peptide mapping.

Other common post-translational modifications, including oxidation and deamidation, were also detected through peptide mapping. These modifications were classified as mild quality attributes due to their potential to alter structure, stability or function. Specifically, deamidation at HC N55 and N388 and oxidation at M256 and M432 were identified as CQAs and were carefully controlled during biosimilar approval.

In this study, comparable oxidation levels were observed across all four products. HC M252 and HC M432, located at the CH2-CH3 interface, exhibited the highest oxidation levels among the residues under unstressed conditions. This finding aligns with previous studies [38–40], which have shown that these residues are particularly susceptible to oxidation. Oxidation of these Met residues can lead to a loss of conformational stability and reduced interaction with the FcRn [34], which in turn shortens in vivo half-life.

Additionally, degradation at the N388/N393 sites was consistent across all rituximab products. The level of succinimide at HC N55 showed slight differences (1–2 %) (Ruxience, Truxima > Riabni, Rituxan), and deamidation followed the same trend, albeit to a lesser degree with less than 1 % variation. In contrast, significant variations were observed in the accumulation of succinimide intermediates at HC N329 (Ruxience > Riabni > Truxima > Rituxan), a modification rarely reported in biosimilar approval documentation. Overall, all the rituximab biosimilars and the reference product displayed higher succinimidation than the NIST mAb (1.44 %) that shares the same tryptic peptide, VSNK. Notably, none of the three rituximab biosimilars, the reference product, or NIST mAb exhibited deamidation at N329. Their high succinimidation observed could be attributed to the formulations of the rituximab products, which are stored at slightly acidic pH values (Ruxience at pH 5.8, and Rituxan, Truxima, Riabni at pH 6.5) to minimize degradation [41]. Additionally, in terms of the process, the low pH-peptide mapping method employed in this study helped minimize deamidation artifact, and facilitated efficient mAb digestion [42], thereby contributing to the significant accumulation of succinimide intermediates [43]. Although deamidation and succinimidation at N329 is reported to potentially decrease ADCC activity [44], Ruxience, which exhibits the highest succinimidation at N329, also shows the highest ADCC effect among the rituximab products. This suggests that a succinimidation level around 10 % may not be critical for ADCC activity. But still, from a manufacturability perspective, it would be beneficial to carefully evaluate its susceptibility to deamidation or succinimidation.

In contrast, HC N280/290, HC N365 and LC N136/N137 are not considered as deamidation hot spots, even though N136/N137 contained up to total 1 % of deamidation and succinimidation. This is because these asparagine residues are in a β-sheet structure area, where it is not accessible to water. During the digestion process, amino acid modifications can occur spontaneously, since tryptic digestion accelerates the rate of modifications due to enhanced accessibility and flexibility of the degradation site in the tryptic peptides compared to the intact native protein [45]. Therefore, degradation on β-sheet structure is expected to occur only after tryptic digestion [45,46]. The degradation extents at these residues in NIST mAb were comparable to those observed in rituximab samples, with 0.27 % of succinimidation at HC N279/289, and 0.50 % and 0.97 % deamidation + succinimidation at HC N364 and LC N136/137, respectively.

Given that the biosimilars analyzed in this study are FDA-approved and have been clinically validated to have no clinically meaningful differences from the reference product, the structural differences observed—within the range detected across these rituximab products—are unlikely to impact clinical outcomes. Therefore, although the advanced analytical tools enable the detection of low-abundance glycan species, their individual contributions to clinical performance are expected to be minimal and likely offset by compensatory effects from other glycan attributes.

A limitation of this study is the restricted number of lots analyzed per product. In this study, three independent lots of each therapeutic were analyzed. These were commercially available, FDA-approved products obtained through the University of Michigan hospital pharmacy. Procurement was constrained by the availability of commercial lots at the time of acquisition. As a result, the statistical power for formal hypothesis testing and multiple-comparison analyses is inherently limited, and our findings should be interpreted with this constraint in mind. Nevertheless, given that these products are manufactured under stringent QA/QC systems ensuring consistent quality across lots, and that all experiments were conducted under strictly controlled and identical conditions, this study strengthens the comparability across products and provides reproducible, descriptive insights.

Stakeholders recognize that biotherapeutics inherently exhibit heterogeneity, and it is critical to define acceptable ranges of this heterogeneity. Urru et al. report that no safety signal emerged in association with the use of a specific biosimilar nor with the practice of switching between different biosimilars or between rituximab reference and biosimilar in clinical [47]. Therefore, our comprehensive analysis is expected to establish the acceptable ranges of differences in both qualitative and quantitative composition. Moreover, our integrated analytical approach—incorporating intact MS, released glycan assays, and peptide mapping—can foster productive discussions among stakeholders and contribute to enhancing current practices for analyzing PTMs in biosimilarity assessments. As we continue to expand our dataset with additional approved therapeutic mAb references and biosimilars, we aim to provide industry, regulatory, and academic stakeholders with deeper insights to support future mAb development.

## Supplementary Material

Supplementary Information

## Figures and Tables

**Table 1:** Identified variants from the deglycosylated rituximab from unsharpened and sharpened deconvoluted spectra.

Proteoform	Theoretical mass (Da)	Name	Measured mass (Da)	Relative Intensity (%)	Measured mass (Da)	Relative Intensity (%)
			Unsharpened spectra		Sharpened spectra	
			
mAb (Reference)	144,186.6	Rituxan	144,192.9 ± 1.2	79.74 ± 0.20	144,189.0 ± 0.4	44.65 ± 2.79
		Riabni	144,194.5 ± 0.5	79.46 ± 0.61	144,189.5 ± 1.0	46.44 ± 0.36
		Ruxience	144,194.8 ± 0.4	65.02 ± 1.15	144,189.1 ± 0.1	37.75 ± 0.92
		Truxima	144,194.4 ±0.4	78.86 ±0.37	144,188.6 ± 0.6	45.68 ± 2.33
mAb, -pyroGlu	144,203.8	Rituxan	ND		144,201.6 ± 0.3	46.38 ± 0.65
		Riabni	ND		144,203.9 ± 1.4	46.20 ± 0.44
		Ruxience	ND		144,202.6 ± 0.6	39.83 ± 0.32
		Truxima	ND		144,203.2 ± 1.0	45.76 ± 1.93
mAb, +Lys	144,314.8	Rituxan	ND		ND	
		Riabni	144,318.5 ± 1.4	1.17 ± 1.17	144,319.5 ± 5.7	1.04 ± 0.56
		Ruxience	144,321.7 ± 0.4	12.50 ± 0.67	144,322.5 ± 0.7	7.92 ± 0.48
		Truxima	ND		ND	
mAb, +2Lys	144,443	Rituxan	ND		ND	
		Riabni	ND		ND	
		Ruxience	144,451.7 ± 0.3	3.70 ± 0.38	144,450.8 ± 0.3	2.67 ± 0.29
		Truxima	ND		144,431.2 ± 1.7	0.16 ± 0.01
mAb, +2Hex	144,511	Rituxan	144,517.6 ± 0.3	7.43 ± 0.12	144,517.5 ± 4.2	3.79 ± 0.19
		Riabni	144,517.5 ± 0.1	7.51 ± 0.68	144,517.4 ± 2.4	4.33 ± 0.64
		Ruxience	144,517.0 ± 0.3	7.76 ± 0.22	144,513.2 ± 0.4	4.44 ± 0.19
		Truxima	144,517.8 ± 0.3	8.05 ± 0.44	144,519.3 ± 1.0	4.43 ± 0.14
mAb, +2Hex, Lys	144,639.2	Rituxan	ND		ND	
		Riabni	ND		ND	
		Ruxience	144,641.3 ± 1.0	1.91 ± 0.06	144,642.0 ± 0.1	1.11 ± 0.08
		Truxima	ND		ND	
mAb, -Gly	144,128.6	Rituxan	ND		144,130.3 ± 1.5	0.33 ± 0.01
		Riabni	ND		144,131.3 ± 0.5	0.22 ± 0.09
		Ruxience	ND		144,134.9 ± 1.0	0.45 ± 0.05
		Truxima	ND		144,136.2 ± 1.9	0.56 ± 0.19

The term ‘mAb (Reference)’ refers to deglycosylated rituximab with 4 N-terminal pyroGlu formation at both HC and LC and 2C-terminal Lys clipping at HC. Lot N = 3; Shown is mean ± standard deviation. Observed masses and relative intensities were measured by intact MS and calculated using ProteinMetrics software.

**Table 2:** Relative percentage contribution of each glycan type for the rituximab products as identified by release glycan (Instant PC).

Glycan common name	Classification	Instant PC labeled MW (Da)	Rituxan		Riabni		Ruxience		Truxima	
			Avg Normalized % area	StDev	Avg Normalized % area	StDev	Avg Normalized % area	StDev	Avg Normalized % area	StDev
			
G0	Complex, afucosylated	1577.63	1.607	0.648	0.897	0.051	3.193	0.275	0.580	0.036
G0-GN (M3G0)	Hybrid, (monoantennary)	1374.55	0.113	0.049	0.087	0.006	ND		0.097	0.015
G0F	Complex, fucosylated	1723.69	41.51	6.404	36.25	1.726	46.38	3.146	41.90	0.971
G0F-GN (M3G0F)	Hybrid, (monoantennary)	1520.61	0.567	0.248	0.41	0.066	0.170	0.01	0.980	0.066
G1	Complex, afucosylated	1739.69	1.357	0.276	0.903	0.057	1.677	0.091	0.190	0.010
G1F	Complex, fucosylated	1885.74	41.4	5.199	46.7	0.921	37.520	2.391	39.360	0.718
G1F-GN (M3G1F)	Hybrid, (monoantennary)	1682.67	1.067	0.183	N/D		ND		1.630	0.075
G1F-GN + NANA (M3G1FS1)	Hybrid, (monoantennary)	1973.76	0.357	0.006	0.23	0.010	ND		0.680	0.053
G1FS1	Complex, fucosylated	2176.84	0.237	0.057	0.263	0.021	1.867	0.092	0.210	0.020
G1F + GN (G1FB)	Complex, fucosylated (triantennary)	2088.82	0.123	0.025	0.310	0.020	0.14	0.010	0.200	0.010
G2	Complex, afucosylated	1901.74	0.357	0.006	0.230	0.010	0.183	0.025	ND	
G2F	Complex, fucosylated	2047.8	7.633	2.098	8.830	0.487	5.393	0.672	6.937	0.236
G2FS1	Complex, fucosylated	2338.89	1.027	0.384	1.083	0.097	1.437	0.138	0.953	0.09
G2FS2	Complex, fucosylated	2629.99	0.550	0.279	0.357	0.050	1.403	0.080	0.513	0.065
Man3	High mannose	1171.48	ND		ND		ND		0.07	0.014
Man5	High mannose	1495.58	1.300	0.350	1.327	0.051	0.587	0.035	2.993	0.133
Man6	High mannose	1657.63	0.253	0.035	0.513	0.049	ND		1.113	0.035
Man7	High mannose	1819.69	0.100	0.026	0.160	0.026	0.023	0.006	0.243	0.038
M5G0F/M4G1F	Hybrid, (monoantennary)	1844.72	ND		0.747	0.055	ND		0.617	0.049
M4G1FS1	Hybrid, (monoantennary)	2135.81	ND		0.31	0.010	ND		0.3	0.017
M5G1F	Hybrid, (monoantennary)	2006.77	0.297	0.05	0.300	0.010	ND		0.303	0.006
M5G1FS1	Hybrid, (monoantennary)	2297.87	0.107	0.055	0.090	0.010	ND		0.153	0.021

Glycans were identified using the Protein Metrics released glycan analysis workflow. Lot *N* = 3.

**Table 3:** Relative percentage contribution of each glycan type for the rituximab products as identified by peptide mapping.

Glycans	Glycan common name	Classification	MW (Da)	Rituxan		Riabni		Ruxience		Truxima
				Avg XIC area summed	StDev	Avg XIC area summed	StDev	Avg XIC area summed	StDev	Avg XIC area summed	StDev
				
HexNAc (1)	-	afucosylated	203.0794	0.089	0.049	0.068	0.014	0.021	0.018	0.080	0.030
HexNAc(l)Fuc (1)	-	-	349.1373	0.022	0.012	0.042	0.019	0.016	0.016	0.017	0.010
HexNAc(2)Hex(2)Fuc (1)	-	-	876.3223	0.011	0.011	0.016	0.014	0.023	0.004	0.029	0.008
HexNAc(3)Hex (2)	-	afucosylated	933.3438	0.018	0.008	ND		0.029	0.008	ND	
HexNAc(3)Hex(2)Fuc (1)	-	hybrid	1079.4017	0.264	0.031	0.235	0.010	0.168	0.085	0.244	0.025
HexNAc(4)Hex (3)	Go	afucosylated	1298.476	1.833	0.624	0.954	0.241	3.153	0.514	0.583	0.067
HexNAc(3)Hex (3)	Go-GN (M3G0)	hybrid, afucosylated	1095.3966	0.208	0.044	0.148	0.032	0.097	0.019	0.136	0.026
HexNAc(4)Hex(3)Fuc (1)	G0F	-	1444.5339	42.967	6.536	37.500	2.234	46.900	5.522	42.433	1.950
HexNAc(3)Hex(3)Fuc (1)	GoF-GN (M3G0F)	hybrid	1241.4545	1.133	0.167	0.918	0.074	0.593	0.153	1.343	0.127
HexNAc(4)Hex (4)	G1	afucosylated	1460.5288	0.667	0.187	0.552	0.112	1.197	0.327	0.307	0.034
HexNAc(3)Hex (4)	G1-GN	hybrid, afucosylated	1257.4494	0.058	0.010	0.024	0.005	0.014	0.008	0.075	0.013
HexNAc(5)Hex (4)	G1B	afucosylated	1663.6082	0.035	0.016	0.047	0.021	0.033	0.013	0.047	0.012
HexNAc(4)Hex(4)Fuc (1)	G1F	-	1606.5867	38.633	4.120	44.633	0.907	35.533	1.823	37.533	0.862
HexNAc(3)Hex(4)Fuc (1)	G1F-GN (M3G1F)	hybrid	1403.5073	0.559	0.014	0.379	0.034	0.173	0.055	1.097	0.159
HexNAc(5)Hex(4)Fuc (1)	G1FB	bisecting GN	1809.6661	0.117	0.024	0.235	0.018	0.144	0.022	0.205	0.011
HexNAc(4)Hex (5)	G2	afucosylated	1622.5816	0.184	0.093	0.183	0.091	0.188	0.099	0.072	0.003
HexNAc(4)Hex(5)Fuc (1)	G2F	-	1768.6395	7.173	1.987	8.363	0.558	4.587	1.046	6.473	0.436
HexNAc(5)Hex(5)Fuc (1)	G2F + GN (G2FB)	bisecting GN	1971.7189	0.046	0.014	0.080	0.013	0.035	0.011	0.047	0.014
HexNAc(4)Hex(6)Fuc (1)	G2F + gal1	α-Gal	1930.6923	0.074	0.031	0.046	0.008	0.023	0.016	0.063	0.012
HexNAc(4)Hex(6)Fuc(1)NeuAc (1)	G2FS1 + gal1	α-Gal, sialylated	2221.7878	0.022	0.014	ND		ND		ND	
HexNAc(2)Hex (3)	Man3	high mannose	892.3172	ND		ND		0.011	0.015	0.204	0.136
HexNAc(2)Hex(3)Fuc (1)	Man3F	high mannose	1038.3751	0.020	0.002	ND		ND		0.034	0.008
HexNAc(2)Hex (4)	Man4	high mannose	1054.3700	0.018	0.007	0.016	0.009	ND		0.088	0.026
HexNAc(2)Hex (5)	Man5	high mannose	1216.4229	1.120	0.219	1.053	0.148	0.334	0.093	2.663	0.311
HexNAc(2)Hex (6)	Man6	high mannose	1378.4757	0.222	0.027	0.174	0.021	0.053	0.019	0.555	0.042
HexNAc(2)Hex (7)	Man7	high mannose	1540.5285	0.093	0.026	0.059	0.015	0.041	0.017	0.208	0.030
HexNAc(2)Hex (8)	Man8	high mannose	1702.5813	0.055	0.006	0.085	0.030	0.069	0.020	0.102	0.027
HexNAc(3)Hex (5)	M5Go	hybrid, afucosylated	1419.5022	0.013	0.006	0.031	0.013	ND		0.063	0.006
HexNAc(3)Hex(5)Fuc (1)	M5G0F/M4G1F	hybrid	1565.5601	0.091	0.013	0.215	0.021	ND		0.223	0.022
HexNAc(3)Hex (6)	M5G1	hybrid, afucosylated	1581.5551	0.028	0.016	0.080	0.022	ND		0.109	0.020
HexNAc(3)Hex(6)Fuc (1)	M6GoF/M5G1F	hybrid	1727.6130	0.111	0.021	0.257	0.022	ND		0.230	0.030
HexNAc(4)Hex(4)NeuAc (1)	G1S1	afucosylated, sialylated	1751.6242	0.013	0.012	ND		0.062	0.019	ND	
HexNAc(3)Hex(4)NeuAc (1)	G1-GN + NeuAc (M3G1S1)	hybrid, afucosylated, sialylated	1548.5448	0.029	0.007	ND		ND		0.043	0.006
HexNAc(4)Hex(4)NeuAc(1)Fuc (1)	G1FS1	sialylated	1897.6821	0.727	0.208	0.676	0.056	2.023	0.539	0.586	0.055
HexNAc(3)Hex(4)NeuAc(1)Fuc (1)	G1F-GN + NeuAc (M3G1FS1)	hybrid, sialylated	1694.6027	0.291	0.063	0.099	0.002	0.045	0.006	0.835	0.099
HexNAc(4)Hex(5)NeuAc (1)	G2S1	afucosylated, sialylated	1913.6770	0.021	0.009	ND		0.127	0.053	ND	
HexNAc(4)Hex(5)NeuAc (2)	G2S2	afucosylated, sialylated	2204.7724	ND		ND		0.023	0.002	ND	
HexNAc(4)Hex(5)Fuc(1)NeuAc (1)	G2FS1	sialylated	2059.7349	1.703	0.614	1.673	0.071	2.073	0.710	1.607	0.162
HexNAc(4)Hex(5)Fuc(1)NeuGc (1)	G2FS1 (NeuGc)	sialylated	2075.7298	0.020	0.014	ND		0.062	0.024	ND	
HexNAc(4)Hex(5)Fuc(1)NeuAc (2)	G2FS2	sialylated	2350.8304	0.856	0.328	0.528	0.051	1.878	0.272	0.925	0.172
HexNAc(4)Hex(5)Fuc(1)NeuAc(1)NeuGc (1)	G2FS2 (NeuAc+NeuGc)	sialylated	2366.8253	0.033	0.033	ND		0.147	0.089	0.011	0.006
HexNAc(3)Hex(5)NeuAc (1)	M4G1S1	hybrid, afucosylated, sialylated	1710.5977	0.022	0.008	0.028	0.003	ND		0.050	0.005
HexNAc(3)Hex(5)Fuc(l)NeuAc (l)	M4G1FS1	hybrid, sialylated	1856.6556	0.063	0.027	0.228	0.022	0.013	0.007	0.210	0.044
HexNAc(3)Hex(6)NeuAc (1)	M5G1S1	hybrid, afucosylated, sialylated	1872.6505	0.028	0.006	0.061	0.009	ND		0.083	0.009
HexNAc(3)Hex(6)Fuc(1)NeuAc (1)	M5G1FS1	hybrid, sialylated	2018.7084	0.307	0.144	0.286	0.020	0.053	0.022	0.340	0.027

Glycans were identified using the Protein Metrics PTM workflow. Lot *N* = 3.

**Table 4:** Summary of identified tryptic peptides, including pyroGlu formation and Lys truncation in rituximab samples (Lot *N* = 3).

Variable position protein		Sequences	Rituxan (%)	Riabni (%)	Ruxience (%)	Truxima (%)

Heavy Chain	1	-.qVQLQQPGAELVK.P	1.15 ± 0.27	1.23 ± 0.06	1.03 ± 0.10	1.03 ± 0.06
	Gln→pyroGlu					
	-17.0265	-.qVQLQQPGAELVKPGASVK.M	98.44 ± 0.26	98.64 ± 0.07	98.23 ± 0.13	98.63 ± 0.04
	451	K.SLSLSPGk.-	99.03 ± 0.47	98.90 ± 0.46	91.50 ± 4.85	99.37 ± 0.06
	Lys-loss					
	-128.0950					
Light Chain	1	-.qIVLSQSPAILSASPGEKV	86.90 ± 0.85	90.80 ± 0.81	74.13 ± 4.92	84.27 ± 0.74
	Gln→pyroGlu					
	-17.0265					

Data are presented as mean ± standard deviation.

**Table 5:** Summary of ADCC activity and FcRγIIIa binding affinity among rituximab products.

	ADCC reporter assay		FcRγIIIa binding assay	
	EC_50_ (ng/mL)	95 % Confidence Interval	IC_50_ (μg/mL)	95 % Confidence Interval

**Rituxan**	9.30	4.86–15.86	10.33	8.84–12.12
**Riabni**	13.58	10.25–18.06	14.08	11.64–17.26
**Ruxience**	6.06	4.56–7.78	9.51	6.59–14.83
**Truxima**	9.09	6.21–12.89	11.48	9.69–13.66

Mean values from 3 technical replicates.

## Data Availability

Data will be made available on request.

## Appendix A. Supplementary data

Supplementary data to this article can be found online at https://doi.org/10.1016/j.ijbiomac.2025.149062.
