# Supplementary material for: Multimodal mass spectrometric characterization of structural microheterogeneity in rituximab reference and biosimilars
Source: Int J Biol Macromol. Author manuscript; Available in PMC 2026 Feb 10. (PMC12889889; doi:10.1016/j.ijbiomac.2025.149062)
Supplement: Supplementary Information [file NIHMS2128865-supplement-Supplementary_Information.docx]

Supplementary Material for

**Multimodal Mass Spectrometric Characterization of Structural Microheterogeneity in Rituximab Reference and Biosimilars**

Y. Na *et al.*

*Corresponding author. Email: [annaschw@umich.edu](mailto:annaschw@umich.edu)

**This file includes:**

Figure. S1 to S11

**Figure S1. Total ion chromatogram (TIC) of rituximab products.** Intact MS TIC chromatograms of untreated (black) and PNGase F-treated (pink) rituximab acquired under native mass spectrometry conditions.


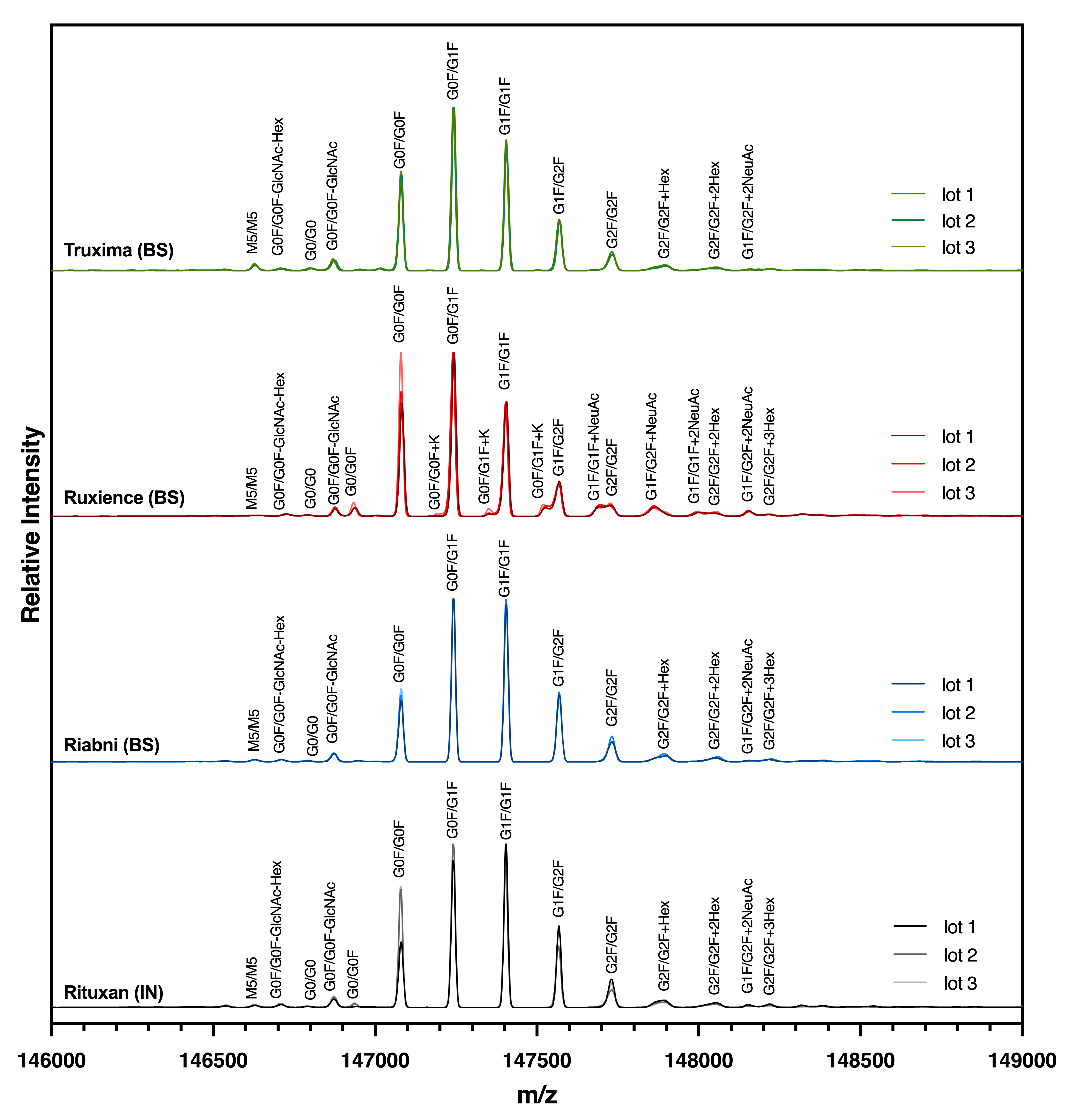


**Figure S2. Deconvoluted intact mass spectra of fully glycosylated rituximab products.** Deconvoluted mass spectrum of fully glycosylated rituximab with sharpened peak data. Glycans and PTMs were identified using the Protein Metrics Intact MS workflow.

**Figure S3. Deconvoluted intact mass spectra of deglycosylated rituximab products.** Deconvoluted mass spectra of deglycosylated rituximab with (A) sharpened and (B) unsharpened peak data. Glycans and PTMs were identified using the Protein Metrics Intact MS workflow.

**Figure S4. MS1 Spectra from LC-MS/MS of the doubly charged tryptic peptide DTLM256ISR.** (A) MS1 spectrum of doubly charged DTLM^256^ISR wild-type precursor at 16.2 min. Observed monoisotopic m/z was m/z 418.221. (B) MS1 spectrum of doubly charged dethiomethylated precursor peptide on M256 at 12.2 min. Observed monoisotopic m/z was 394.221. (C) MS1 spectrum of doubly charged oxidated precursor peptide on M256 at 14.8 min. Observed monoisotopic m/z was 426.217.

**Figure S5. Characterization of product ions in the MS/MS spectrum of the doubly charged tryptic peptide DTLM256ISR.** (A) Wild-type peptide. (B) Dethiomethylation at M256, confirmed by a -48 Da mass shift in the corresponding insets, with diagnostic shifts in the y4, b4, y5, b5, y6, and b6 fragment ions confirming the modification. (C) Oxidation at M256, evidenced by a +16 Da mass shift, with corresponding shifts in the y4, b4, y5, y6, and b6 ions supporting the assignment.

**Figure S6. MS1 Spectra from LC-MS/MS of the doubly charged tryptic peptide GLEWIGAIYPGN^55^GDTSYNQK.** (A) MS1 spectrum of doubly charged GLEWIGAIYPGNGDTSYN^55^QK wild-type precursor at 22.6 min. Observed monoisotopic m/z was m/z 1092.020. (B) MS1 spectrum of doubly charged diamidated precursor peptide on N55 at 22.8 min. Observed monoisotopic m/z was 1092.518. (C) MS1 spectrum of doubly charged succinimidated precursor peptide on N55 at 22.8 min. Observed monoisotopic m/z was 1083.314.

**Figure S7. Characterization of product ions in the MS/MS spectrum of the doubly charged tryptic peptide GLEWIGAIYPGN^55^GDTSYNQK.** (A) Wild-type peptide. (B) Deamidation at N55, identified by a +1 Da mass shift in the corresponding insets, with diagnostic shifts in the y9, y10, y11, y12, y13, and y15 fragment ions confirming the modification. (C) Succinimidation at N55, evidenced by a -17 Da mass shift, with corresponding shifts in the y11, y12, y13, y14, and y15 fragment ions supporting the assignment.

**Figure S8.** **Comparison of FcRγIIIa binding affinity between intact and deglycosylated rituximab products.** FcRγIIIa (V158 variant) binding was measured for (A) Rituxan, (B) Riabni. (C) Ruxience, and (D) Truxima using the Lumit immunoassay. All deglycosylated samples showed a marked decrease in binding relative to their intact counterparts, confirming the dependence of FcRγIIIa interaction on Fc N-glycans. Data represent mean ± standard deviation of technical triplicates (N=3); dose response curve was fitted with a 4-parameter model using GraphPad Prism® software.

**Figure S9. Comparison of ADCC activity between intact and deglycosylated rituximab products.** ADCC activity was measured for (A) Rituxan, (B) Riabni, (C) Ruxience, and (D) Truxima using Wil2-S target cells and Jurkat effector cells expressing the high-affinity V158 variant. All deglycosylated samples lost detectable ADCC activity, highlighting the critical role of Fc N-glycans in ADCC function. Fold of induction was calculated by dividing RLU (induced-background) by the RLU (no antibody control - background). Data represent mean ± standard deviation of technical triplicates (N=3); dose response curve was fitted with a 4-parameter model using GraphPad Prism® software.

**Figure S10. SEC-UPLC chromatograms of intact and deglycosylated rituximab products detected at 280 nm. (**A) Intact rituximab samples exhibited a single, symmetric monomeric peak at 5.98 min with no detectable aggregation or fragmentation. (B) After PNGase F treatment, deglycosylated rituximab samples showed an additional PNGase F enzyme peak at 6.56 min, while the monomeric mAb peak remained at 5.99 min, indicating that deglycosylation did not induce aggregation or degradation of the antibody.

**Figure S11.  Deconvoluted mass spectra of Nist mAb.** Deconvoluted intact MS spectra for (A) fully glycosylated and (B) deglycosylated NIST mAb. The glycoforms and post-translational modifications (PTMs) identified by Protein Metrics Intact MS workflow are shown.
